# Derlin-1 Regulates Mutant VCP-Linked Pathogenesis and Endoplasmic Reticulum Stress-Induced Apoptosis

**DOI:** 10.1371/journal.pgen.1004675

**Published:** 2014-09-25

**Authors:** Cyong-Jhih Liang, Ya-Chu Chang, Henry C. Chang, Chung-Kang Wang, Yu-Chien Hung, Ying-Er Lin, Chia-Ching Chan, Chun-Hong Chen, Hui-Yun Chang, Tzu-Kang Sang

**Affiliations:** 1 Institute of Biotechnology, Department of Life Science, National Tsing Hua University, Hsinchu, Taiwan; 2 Department of Biological Sciences, Purdue University, West Lafayette, Indiana, United States of America; 3 Institute of Molecular and Genomic Medicine, National Health Research Institutes, Zhunan, Miaoli County, Taiwan; 4 Brain Research Center, National Tsing Hua University, Hsinchu, Taiwan; 5 Institute of Systems Neuroscience, National Tsing Hua University, Hsinchu, Taiwan; Stanford University School of Medicine, United States of America

## Abstract

Mutations in VCP (Valosin-containing protein), an AAA ATPase critical for ER-associated degradation, are linked to IBMPFD (Inclusion body myopathy with Paget disease and frontotemporal dementia). Using a *Drosophila* IBMPFD model, we have identified the ER protein Derlin-1 as a modifier of pathogenic TER94 (the fly VCP homolog) mutants. Derlin-1 binds to TER94 directly, and this interaction is essential for Derlin-1 overexpression to suppress the pathogenic TER94-induced neurodegeneration. Derlin-1 overexpression reduces the elevated ATPase activity of pathogenic TER94, implying that IBMPFD is caused by ATPase hyper-activation. Under physiological condition, Derlin-1 expression is increased upon ER stress to recruit TER94 to the ER. However, in response to severe ER stress, Derlin-1 is required for activating apoptosis to eliminate damaged cells. This pro-apoptotic response is mimicked by Derlin-1 overexpression, which elicits acute ER stress and triggers apoptosis via a novel C-terminal motif (α). As this Derlin-1-dependent cell death is negated by TER94 overexpression, we propose that while Derlin-1 and VCP work cooperatively in ER stress response, their imbalance has a role in removing cells suffering prolonged ER stress.

## Author Summary

We have previously developed a fly model for IBMPFD (inclusion body myopathy with Paget disease and frontotemporal dementia) and demonstrated that specific mutations in VCP gene, a highly conserved ATPase, cause muscle and neuron degeneration by depleting cellular ATP level. Using this model, we show that expression of Derlin-1, an ER membrane protein capable of directly interacting with VCP, restores the normal cellular ATP level and suppresses IBMPFD-like neurodegeneration. As Derlin-1 expression can be induced by tunicamycin (an antibiotic) in experimental systems, our findings may yield new therapeutic strategies for VCP-linked diseases. In addition, we have obtained important insights regarding Derlin-1 function under physiological conditions. ER stress, caused by accumulation of improperly folded proteins, results in increased Derlin-1 expression, which is important for ER stress-induced cell death. We propose that Derlin-1 promotes ER homeostasis through multiple mechanisms. In addition to cooperating with VCP to extract improperly folded proteins from the ER, elevated Derlin-1 expression removes cells suffering from irreparable ER stress, thus preventing these damaged cells from further harming the organisms.

## Introduction

Valosin-containing protein (VCP), a highly conserved AAA (ATPase associated with various cellular activities) ATPase, has been implicated in proteasomal degradation [1], cell cycle control [2], membrane fusion [3], [4], transcription activation [5], and endoplasmic reticulum (ER)-associated degradation (ERAD) [6], [7]. To achieve this functional plasticity, VCP cooperates with a number of cofactors/adaptors to process specific substrates. For instance, VCP, with p47, promotes Golgi reassembly at the end of mitosis [8], whereas VCP, along with Ufd1/Npl4, expels misfolded protein from the ER [9].

Given its importance in various cellular pathways, it is not surprising that mutations in VCP cause diseases. Indeed, specific mutations in VCP have been linked to IBMPFD (Inclusion body myopathy with Paget disease and frontotemporal dementia), an autosomal dominant, multi-system degenerative disorder [10]. VCP contains two ATPase domains (D1 and D2), preceded by the N-terminal CDC48 and L1 (first linker) domains. These IBMPFD-associated mutations are clustered in the N-terminal portion of VCP, and have not been found in the major ATPase domain D2 [11], suggesting that they are not loss-of-function alleles. In support of this, biochemical studies have shown that IBMPFD-linked VCP mutants still preserves ATPase activity [12]–[14], and we have genetically demonstrated that three of these disease alleles (R155H in CDC48 domain, R191Q in L1 domain, and A232E in L1-D1 junction) are dominant active mutations [15].

A number of mechanisms have been proposed to account for the pathogenesis of IBMPFD. Cultured cells expressing VCP^R155H^ showed an accumulation of misfolded substrates, suggesting that this common disease mutant causes IBMPFD by disrupting ERAD [16]. In transgenic and knock-in mouse models, VCP^R155H^ expression caused an accumulation of autophagosome-associated proteins, implying that impaired autophagy is a cause for IBMPFD [17], [18]. TDP-43 (TAR-DNA-binding protein 43)-containing aggregates have also been linked to VCP disease mutant-induced cytotoxicity [17], [19], although whether the accumulation of these proteinaceous structures is a direct cause of IBMPFD remains unclear [20]. We have established a *Drosophila* IBMPFD model, in which muscular and neuronal tissue-specific expression of pathogenic TER94 mutants (the fly VCP homolog carrying mutations analogous to those implicated in IBMPFD) caused degeneration [15]. Pathogenic TER94 mutants exhibited elevated ATPase activities [12]–[14], suggesting that depletion of cellular ATP contributes to IBMPFD pathogenesis. As VCP acts in numerous cellular processes, it is possible that VCP mutants cause IBMPFD via multiple distinct mechanisms.

Here we show that overexpression of Derlin-1, an interacting partner of VCP in the ERAD pathway, inhibits the elevated ATPase activities of pathogenic TER94 mutants and suppresses the neurodegenerative defects. Der1 family proteins have emerged as an important component of the ERAD pathway. Mammalian Derlin-1 participates in the retrotranslocation of major histocompatibility complex class I protein [21], [22], and Derlin-2 and Derlin-3, two other human paralogs, have been implicated in ERAD [23]. Structural analyses indicate that Derlin-1 spans the ER membrane either four [21], [24], [25] or six [26] times, consistent with the notion that Derlin-1 functions as a channel for substrate passage through the ER membrane [21], [27]–[29]. Both yeast and human Derlin-1 homologs contain the so-called SHP box in their cytosolic C-terminal tails, which bind to respective AAA ATPases [24], [26], [30].

We show that overexpression of Derlin-1 alone impairs ERAD, activates UPR (Unfolded Protein Response), and causes apoptosis. All these Derlin-1 overexpression defects are suppressed by increased TER94 expression, suggesting that an imbalance between Derlin-1 and TER94 levels is detrimental to cells. As Derlin-1 expression is elevated in response to ER stress, we propose that while Derlin-1 participates in the retrotranslocation of misfolded proteins, prolonged ER stress activates the cytotoxic function of Derlin-1 to prevent these damaged cells from harming the organismal health.

## Results

### Derlin-1 modulates TER94 overexpression induced neurodegeneration

**Figure 1:**
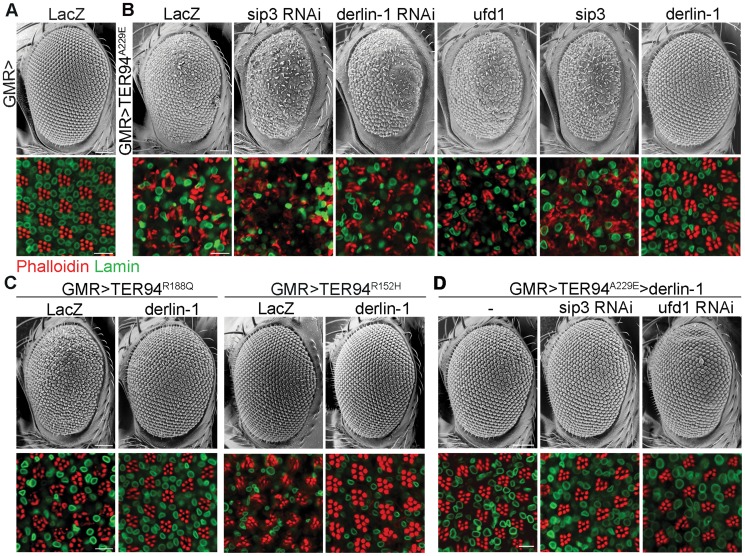
Derlin-1 modifies the neurodegeneration associated with the pathogenic TER94 mutants. Scanning electron micrographs (SEM) of adult eyes (upper rows) and confocal sections of retina (lower rows) stained with phalloidin (red) and anti-Lamin antibody (green). (A) The compound eye of *GMR>LacZ* exhibits a highly ordered structure composed of approximately 750 facets known as ommatidia. The organization of underlying photoreceptors is revealed by phalloidin, which stains the light-sensing rhabdomeres. Lamin antibody marks the nuclear envelopes. (B) Eye phenotypes from *GMR>TER94^A229E^* with RNAi-mediated knockdown of *sip3* and *derlin-1*, and with overexpression of *ufd1*, *sip3*, and *derlin-1*. (C) Eye phenotypes of two additional TER94 disease mutants, *GMR>TER94^R188Q^* and *GMR>TER94^R152H^*, with or without *derlin-1* co-expression. (D) Eye phenotypes from *GMR>TER94^A229E^>derlin-1* with RNAi-mediated knockdown of *sip3* and *ufd1*. All images are collected from 1-day-old adult except *GMR>TER94^R152H^* group in (C), which are 18-day-old adult. For the SEM images, anterior is to the left and dorsal is up. Scale bars: 100 µm (SEM), 10 µm (confocal).

We have reported a *Drosophila* IBMPFD model, in which targeted expression of TER94 disease mutants in muscle and nervous systems recapitulates pathophysiological features of the disease, causing tissue degeneration, inclusion body formation and learning deficit [15]. Because this ATPase cooperates with various cofactors and adaptor proteins, we selected 11 candidate genes from literatures [6], [31], [32] and asked whether disruption of any of these accessory proteins by RNAi (dsRNA-mediated RNA interference) might alter the cytotoxicity of a strong pathogenic mutant TER94^A229E^ (the homologous mutation of VCP^A232E^). We took advantage of the fact that *GMR>TER94^A229E^* flies (expressing *UAS-TER94^A229E^* transgene under the control of the eye-specific *GMR-GAL4* driver) showed rough eyes, and the highly organized architecture of *Drosophila* eye offers an easy and powerful means to detect genetic interactions. Of the RNAi lines tested, knockdown of three genes, namely *derlin-1*, *ufd1*, and *sip3* (the *Drosophila* Hrd1 homolog), exhibited more severe phenotypes (Table S1). Knockdown of Ufd1, a cofactor of VCP ATPase [33], was synthetic lethal with TER94^A229E^. Knockdown of Sip3 and Derlin-1, two ER membrane proteins thought to form a retrotranslocation passage to expel ERAD substrates [27], enhanced the *GMR>TER94^A229E^* eye roughness and the underlying photoreceptor degeneration (Figure 1A and 1B). As these proteins are known to interact with VCP in cultured cells [26], [34], [35], these results demonstrate that this *Drosophila* IBMPFD model is capable of identifying relevant targets.

To test whether overexpression of these genes has complementary effect (i.e. suppression) on the phenotype of VCP pathogenic mutant, *derlin-1*, *ufd1*, and *sip3* cDNAs were overexpressed with *GMR-GAL4* and tested for interaction with *GMR>TER94^A229E^*. Although *ufd1* and *sip3* RNAi enhanced *GMR>TER94^A229E^*, overexpressing either *ufd1* or *sip3* had no apparent effect on the rough eye and the underlying photoreceptor degeneration (Figure 1B). In contrast, *derlin-1* overexpression completely suppressed *TER94^A229E^*-induced eye phenotypes (Figure 1B). Overexpressing *derlin-1* also rescued another two disease mutants *TER94^R152H^* and *TER94^R188Q^* (Figure 1C), demonstrating that elevated Derlin-1 expression counters the adverse effects of different IBMPFD mutants. Furthermore, this rescue of pathogenic TER94 by Derlin-1 does not appear to require Sip3 and Ufd1, as RNAi knockdown of *sip3* or *ufd1* (Figure S1) showed no effect on the restored *GMR>TER94^A229E^>derlin-1* eyes (Figure 1D).

### Direct interaction with TER94 is essential for Derlin-1-dependent suppression

**Figure 2:**
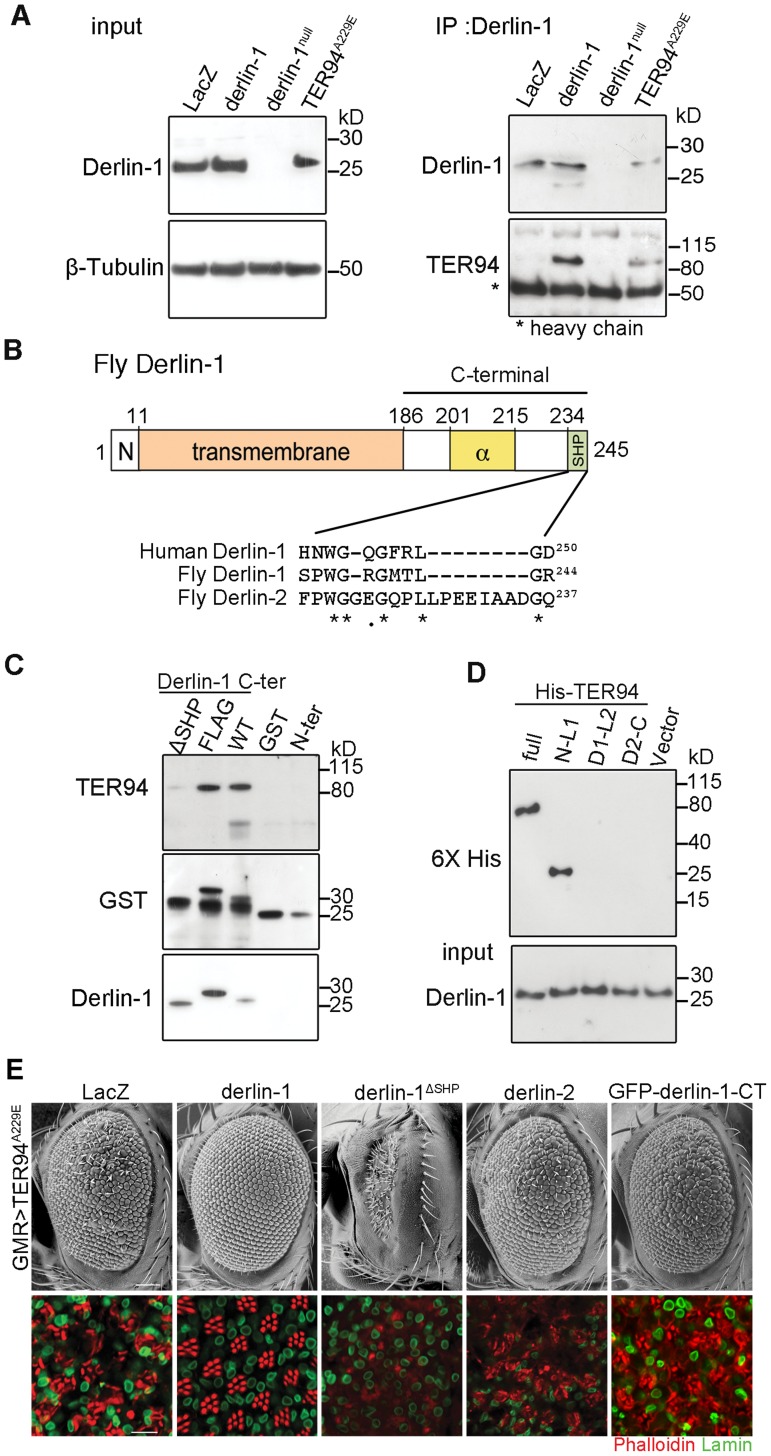
The suppression of TER94^A229E^ by Derlin-1 overexpression requires direct interaction. (A) Western analysis of lysates and anti-Derlin-1 immunoprecipitates from *hs>LacZ; tub-GAL80^ts^* (control, *hs-GAL4* in combination with *tub-GAL80^ts^* to drive LacZ expression; see Materials and Methods), *hs>derlin-1; tub-GAL80^ts^* (derlin-1 expression), *derlin-1^null^*, and *hs>TER94^A229E^; tub-GAL80^ts^* (TER94^A229E^ expression). The lysate (input) blot was detected by anti-Derlin-1 and then stripped to re-probe with anti-β-Tubulin for loading control, whereas the IP blot was probed with anti-VCP and anti-Derlin-1 to detect co-IPed TER94 and Derlin-1, respectively. The band corresponding to Ig heavy chains is indicated by asterisk. (B) A schematic diagram of Derlin-1 domains, depicting the N-terminal cytoplasmic segment (denoted N), the six transmembrane domains (beige box), the α-domain (yellow box) and the C-terminal SHP domain (green box). A ClustalW sequence alignment of the putative C-terminal SHP domains from human and fly Derlin homologs. Identical (asterisks) and similar (dots) residues shared by the homologs are denoted. (C) GST pull-down of TER94 from *GMR>TER94^A229E^* head extract by Derlin-1 truncations or C-terminally FLAG-tagged Derlin-1. The pull-downed TER94 proteins were detected by immunoblotting with anti-VCP (upper panel) antibodies, and the blot was re-probed by anti-GST antibodies (middle panel) and anti-Derlin-1 antibodies (lower panel). GST alone is included as a control. (D) Pull-down of bacterially expressed His-tagged TER94 truncations by GST-Derlin-1 C-terminal fragment. The blot was probed with anti-Derlin-1 antibodies (input), followed by re-probing with anti-6XHis antibodies. Only full-length His-TER94 and His-TER94^N-L1^ interact with the Derlin-1 C-terminal fragment. (E) SEM (upper row) and confocal section of retinas (lower row) from 1-day-old adults with indicated transgenes expressed under *GMR-GAL4* control. The retinas are stained with phalloidin (red) and anti-Lamin antibody (green) to visualize the rhabdomeres and the nuclear envelopes, respectively. Overexpression Derlin-1, but not Derlin-1^ΔSHP^, Derlin-2, or GFP-Derlin-1-CT, suppresses pathogenic TER94^A229E^-induced eye degeneration. Scale bars: 100 µm (SEM), 10 µm (confocal).

Yeast and mammalian Derlin-1 homologs are known to interact with their corresponding VCP homologs through the SHP box [26], [30]. Likewise, Derlin-1 forms a complex with TER94, as a TER94 band was detected in the *hs>derlin-1* (wild-type Derlin-1 driven by a heat shock-inducible GAL4) extracts co-immunoprecipitated (co-IP) with a polyclonal anti-Derlin-1 antiserum (Figure 2A; see Materials and Methods). No TER94 band was seen when Derlin-1 expression was knocked out in a null mutant (*derlin-1^l(2)SH1964^*; herein referred as *derlin-1^null^*), demonstrating the specificity of the co-IP analysis (Figure 2A). This TER94 band was also present in the co-IP of *hs>TER94^A229E^* extracts, suggesting that the pathogenic mutation does not interfere with Derlin-1/TER94 association.

Inspection of *Drosophila* Derlin-1 protein sequence revealed a C-terminal segment with limited similarity to the SHP box (Figure 2B). To ask whether this putative SHP box is responsible for the association of Derlin-1 with TER94, pull-down assays were performed by incubating purified GST-Derlin-1 fusion proteins with fly extract (Figure 2C). While GST alone and the Derlin-1 N-terminal portion were incapable of binding to TER94, the Derlin-1 C-terminal portion was sufficient to pull down both TER94 wild-type (TER94^WT^) and TER94^A229E^ (Figure 2C and Figure S2A). This binding was abolished without the putative SHP box, suggesting that it facilitates the direct association of Derlin-1 C-terminal portion with TER94 (Figure 2C).

To pinpoint the TER94 domains critical for its interaction with Derlin-1 and to test a direct interaction between them, purified His-tagged TER94 fragments were subjected to pull-down assays with GST-Derlin-1 (see Materials and Methods). Similar to the full-length TER94, a truncation with the CDC48 domain and first linker (N-L1) was bound to Derlin-1. In contrast, truncations containing the ATPase domains (D1 and D2) and His alone showed no interaction (Figure 2D). These results demonstrate that the N-terminal portion of TER94 is responsible for interacting directly with Derlin-1.

To test whether this direct interaction is essential for the Derlin-1-mediated suppression of *GMR>TER94^A229E^*, we generated *UAS-derlin-1^ΔSHP^*, which lacks the putative SHP box. Unlike wild-type *derlin-1*, overexpressing *derlin-1^ΔSHP^* failed to suppress *GMR>TER94^A229E^* (Figure 2E), demonstrating that this putative SHP box in Derlin-1 is crucial for its physical and functional interactions with VCP. Consistent with this, overexpressing fly Derlin-2, which contains a more divergent SHP box (interrupted by eight amino acids as aligned by ClustalW; Figure 2B) and does not bind to TER94 (Figure S2B), showed no rescue of *GMR>TER94^A229E^* (Figure 2E).

To ask if the ER association is crucial for Derlin-1 to suppress TER94^A229E^, we generated transgenic lines expressing Derlin-1 C-terminal cytoplasmic tail (aa. 189∼245; including the SHP box) fused to GFP (GFP-Derlin-1-CT). Unlike wild-type Derlin-1, this N-terminal transmembrane domain-deleted construct failed to suppress TER94^A229E^ (Figure 2E), suggesting that besides interacting directly with TER94^A229E^, the ER localization of Derlin-1 is critical for suppressing the disease mutant phenotype.

### Derlin-1 overexpression reduces TER94 ATPase activity

**Figure 3:**
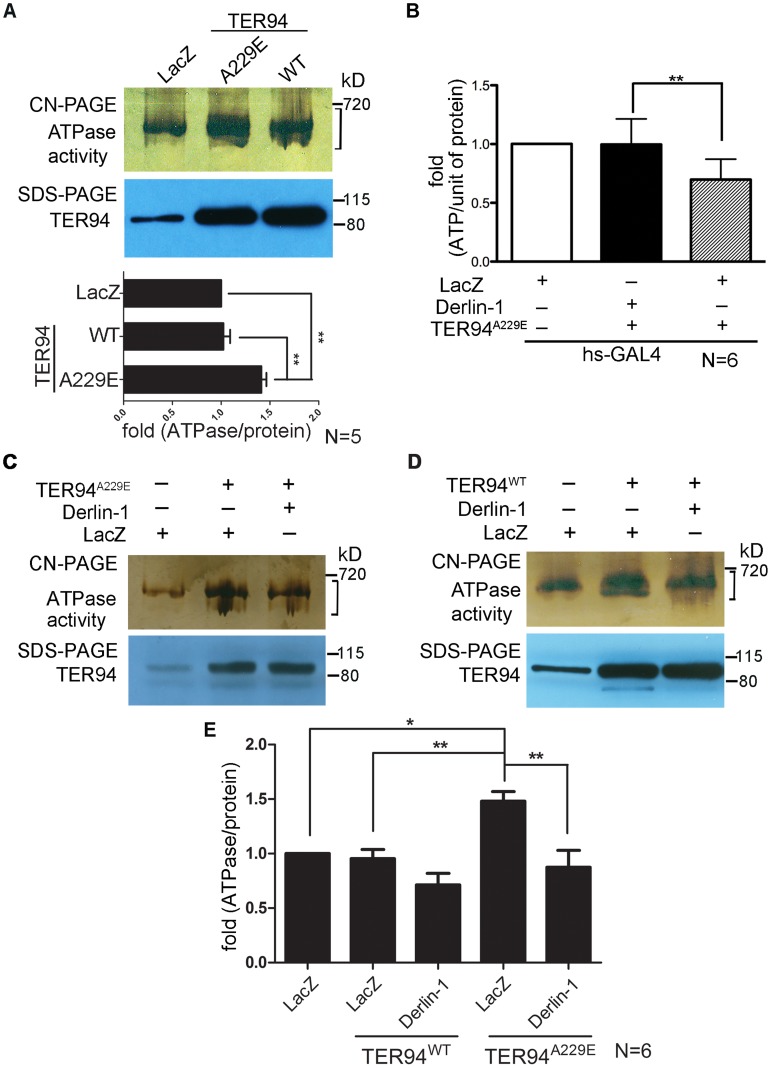
Overexpressing Derlin-1 suppresses the ATPase activity of pathogenic TER94 mutant. (A, C and D) In-gel ATPase activity assay of indicated transgenes driven by *hs-GAL4*; *tub-GAL80^ts^*. The ATPase activity presented with bands intensity (see Materials and Methods) from the clear-native (CN)-PAGE was normalized by TER94 protein levels in SDS-PAGE. A bracket marks the measured bands for this representative CN-PAGE. Quantification of ATPase activities from flies expressing LacZ control and TER94 transgenes in panel A is shown. Values represent the mean± SE from five independent experiments. **p<0.01 (one-way ANOVA with Bonferroni's multiple comparison test). (B) Measurement of cellular ATP levels in flies carrying indicated transgenes driven by *hs-GAL4*. Values represent the means ± SE from six independent experiments (**p<0.01; one-way ANOVA with Bonferroni's multiple comparison test). (E) Measurement of ATPase activities from C and D. Values represent the mean± SE from six independent experiments. *p<0.05; **p<0.01 (one-way ANOVA with Bonferroni's multiple comparison test).

We have shown that TER94^A229E^ expression causes defects in the eye morphology and photoreceptor organization by depleting cellular ATP [15]. To correlate this pathogenic phenotype with the enzymatic properties of TER94 mutant, we performed an in-gel assay to directly measure the ATPase activity of TER94^A229E^. Compared with TER94^WT^, TER94^A229E^ exhibited a ∼40% increase in ATPase activity (Figure 3A), consistent with the notion that this pathogenic TER94 mutant consumes more ATP in vivo.

To understand the mechanism underlying this Derlin-1-mediated suppression, we asked whether Derlin-1 overexpression restores the cellular ATP level in TER94^A229E^-expressing flies. Compared to the *hs>LacZ* control, *hs>TER94^A229E^* flies exhibited a 25% reduction in the cellular ATP level (Figure 3B). In contrast, flies co-expressing Derlin-1 with TER94^A229E^ had similar ATP level as *hs>LacZ* (Figure 3B), suggesting that Derlin-1 suppresses TER94^A229E^ defects by restoring the cellular ATP level.

As Derlin-1 binds to TER94, Derlin-1 overexpression may affect the elevated ATPase activity of disease mutant directly. To test this, we measured the TER94 activity in different genetic backgrounds. Consistent with the reduced cellular ATP level in TER94^A229E^-expressing tissue (Figure 3B), enhanced ATPase activity was observed in this disease mutant (Figure 3C). Compared to TER94^A229E^ alone, TER94^A229E^ in the presence of transient Derlin-1 expression showed a ∼40% reduction in its ATPase activity (Figure 3C and 3E). This Derlin-1-dependent reduction of TER94 ATPase activity is not restricted to disease mutant, as a similar inhibition was observed with TER94^WT^ (Figure 3D and 3E). These observations suggest that elevated Derlin-1 expression restores the cellular ATP level in the IBMPFD model by directly inhibiting the mutant TER94 ATPase activity.

### Derlin-1 recruits TER94 to the ER in ERAD

**Figure 4:**
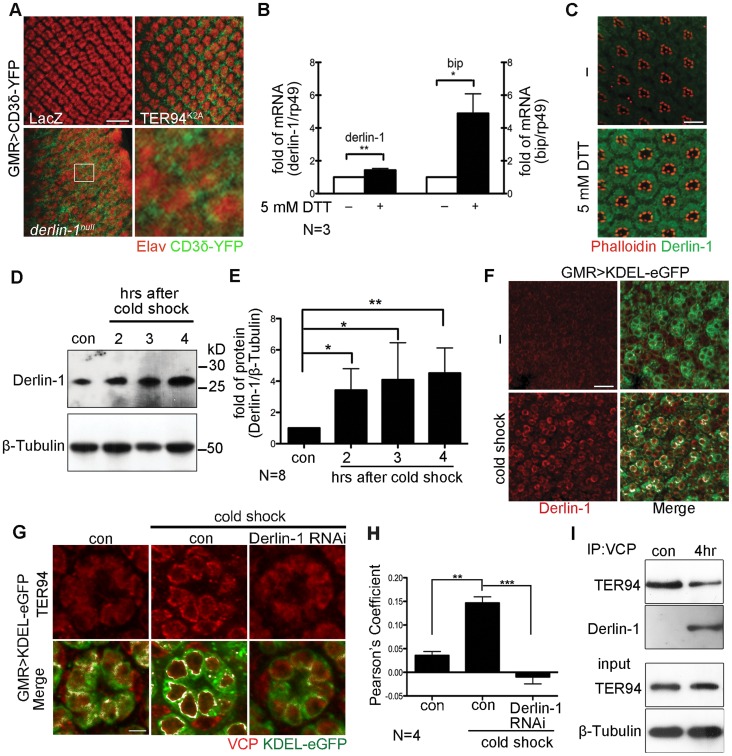
ER stress increases Derlin-1 expression and promotes the recruitment of TER94 to the ER. (A) Confocal images of control *GMR>LacZ*, *GMR>TER94^K2A^*, and *derlin-1^null^* larval eye discs expressing CD3δ-YFP (green). The eye discs are stained with anti-Elav antibodies (red) to label neuronal nuclei. Expression of TER94^K2A^ serves as a positive control for CD3δ-YFP. The boxed region in the *derlin-1^null^* panel is shown at a higher magnification. (B) Quantitative RT-PCR analysis of *derlin-1* and *bip* transcripts from eye discs with (+) and without (−) 5 mM DTT treatment. Results from three independent quantitative RT-PCR experiments, after being normalized to *rp49* levels, are shown in fold change (compared to untreated). Values shown represent mean ± SE. *p<0.05; **p<0.01 (Student's *t*-test). (C) Confocal images of wild-type mid-pupal eyes with and without (−) 5 mM DTT treatment, stained with phalloidin (red) and anti-Derlin-1 (green). (D) Quantitative Western of endogenous Derlin-1 protein levels from flies subjected to 2 hrs cold shock at 0°C. Lysates from wild-type flies (con) and those recovered after the cold shock for the indicated time periods are probed with anti-Derlin-1 antibody. β-Tubulin levels serve as loading control. (E) Results from eight independent experiments in D are shown. Derlin-1 protein levels, normalized to loading controls, are shown in fold change as compared to untreated control. Values shown represent mean ± SE. *p<0.05; **p<0.01 (one-way ANOVA with Bonferroni's multiple comparison test). (F and G) Confocal images of *GMR>KDEL-eGFP* mid-pupal eyes, before (“−“ in F; left panels in G) and after the cold treatment (cold shock), stained with anti-Derlin-1 (F) or anti-VCP (G) antibodies (red). KDEL-eGFP (green) labels the ER, and the co-localization with KDEL-eGFP in merged panels is shown in white. (H) Pearson's co-localization coefficient analyses of images from four independent experiments as in G (see Materials and Methods for details). Cold shock treatment shows enhanced correlation of pixel pairs that label TER94 and the ER in a Derlin-1-dependent manner. Scale bars: 10 µm. (I) Western analysis of lysates and anti-VCP immunoprecipitates from flies treated with (4 hrs) or without (con) cold shock. The IP blot was probed with anti-VCP and anti-Derlin-1 to detect TER94/Derlin-1 complexes. The lysate (input) blot was detected by anti-VCP, and then stripped and re-probed with anti-β-Tubulin for loading control.

While the retrotranslocation of model ERAD substrate is defective in cells expressing TER94^K2A^ (a dominant-negative TER94 mutant; Figure 4A) [15], [36], the significance of VCP/Derlin-1 interaction is not fully understood. To investigate whether fly Derlin-1 participates in ERAD, we monitored the signal from CD3δ-YFP, a well-established fluorescent ERAD substrate [15], [37], in eye discs homozygous for *derlin-1^null^*. While the CD3δ-YFP signal was absent in control, CD3δ-YFP accumulated around the nuclei in *derlin-1^null^* eye disc cells (Figure 4A), indicating that fly Derlin-1, like TER94, is required for ERAD.

To understand the role of fly Derlin-1 in maintaining ER homeostasis, we tested whether its expression responds to ER stress. Treating eye discs with dithiothreitol (DTT), a known ER stressor [38], resulted in an increase of endogenous *derlin-1* mRNA and protein (Figure 4B and 4C). DTT treatment also elevated the mRNA level of *bip*, the ER chaperone commonly serves as the hallmark of UPR [39]. To demonstrate that this Derlin-1 response can be elicited by an independent ER stress other than pharmacological treatments, adult flies were treated with a cold shock (two hours at 0°C), which transiently detains secretory proteins in the ER and activates the UPR response as reported by Xbp1-eGFP [40], [41] (Figure S3). Similar to the DTT treatment, Derlin-1 protein level was increased by the cold shock (Figure 4D and 4E). Moreover, induced Derlin-1 partially co-localized with KDEL-eGFP, an ER-marker (Figure 4F). Together, these results demonstrate that Derlin-1 expression responds to ER stress.

To ask whether TER94 expression, like Derlin-1, responds to ER stress, we measured the TER94 protein level in flies treated with a cold shock. Quantitative Western analyses showed that the level of endogenous TER94 was not altered by the treatment (Figure S4), although the association of TER94 with the ER was noticeably increased (Figure 4G). This co-localization of TER94 with KDEL-eGFP was reduced by *derlin-1* knockdown (Figure 4G), implying that the stress-induced Derlin-1 expression serves to recruit TER94 to the ER. As Derlin-1 knockdown showed a reduced KDEL-eGFP signal, images from four independent experiments were quantified to confirm that the co-localization between TER94 and KDEL was Derlin-1-dependent (Figure 4H). In support of these co-localization studies, co-IP with anti-VCP antibodies showed that the level of Derlin-1/TER94 complexes increased by 4.9 ± 0.98 folds (after normalization with TER94 levels) after cold treatment (Figure 4I). All together, these data suggest that coordinated interaction between Derlin-1 and TER94 not only affects the pathogenesis of IBMPFD disease mutant, but also acts in an ER stressed condition.

### Derlin-1 is required for ER stress-induced caspase activation

**Figure 5:**
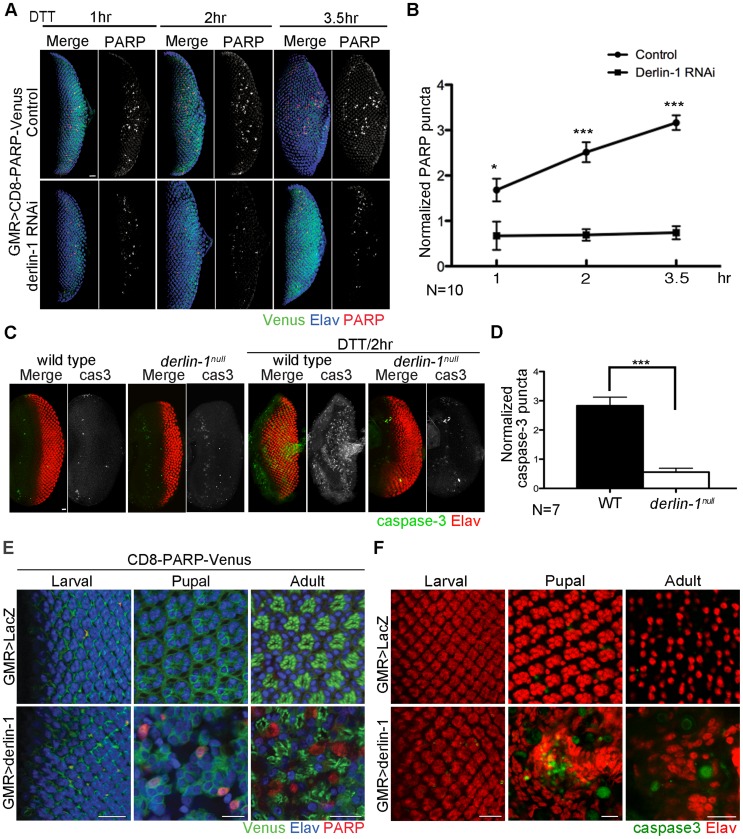
Derlin-1 involves in ER stress-induced caspase activation. (A) Confocal images of *GMR>CD8-PARP-Venus* eye discs treated with 5 mM DTT for 1-, 2- and 3.5-hrs. In the lower row, Derlin-1 expression is reduced by *derlin-1 RNAi*. The eye discs are stained with anti-PARP (red) and anti-Elav (blue) to mark activated caspase activity and neuronal nuclei, respectively. The anti-PARP signals are shown separately from the merged images for comparison. (B) Quantification of anti-PARP puncta from DTT-treated eye discs shown in A. For each eye disc, anti-PARP signals were normalized to the disc size (numbers of PARP puncta/rows of ommatidia), and ten eye discs for each time point was plotted. (C) Confocal images of wild-type and *derlin-1^null^* eye discs stained with anti-Elav (red) and anti-cleaved caspase-3 (green) antibodies. The eye discs in the right panels were treated with 5 mM DTT for 2 hours. The caspase-3 signals are shown separately from the merged images for comparison. (D) Quantification of anti-cleaved caspase-3 puncta as shown in C. Punta numbers were normalized to the disc size as described in B. Seven eye discs were analyzed. Values shown in B and D represent mean ± SE. *p<0.05; ***p<0.001 (Student's t-test). (E) Confocal images of *GMR>LacZ* (upper row) and *GMR>derlin-1* (lower row) eyes at the larval, pupal, and adult stages, stained with anti-Elav antibodies to label photoreceptor nuclei (blue). These eyes carry a membrane-tethered CD8-PARP-Venus (green), and are stained with anti-PARP antibodies (red) to detect caspase-mediated cleavage events. Scale bars: 10 µm. (F) Confocal images of larval, pupal, and adult *GMR>LacZ* (upper row) and *GMR>derlin-1* (lower row) eyes stained with anti-Elav (red) and anti-cleaved caspase-3 (green) antibodies. Scale bars: 10 µm.

Cells suffering from excessive ER stress are often eliminated by apoptotic cell deaths. As Derlin-1 expression is elicited by ER stress, we hypothesize that Derlin-1 has a role in facilitating the elimination of cells enduring prolonged ER stress. To test this, we asked whether *derlin-1* knockdown influences DTT-induced caspase activation. *UAS-CD8-PARP-Venus*, an effector caspase reporter, was used to detect the cleavage events generated by caspase-3-like (DEVDase) activity [42] in larval eye discs. *GMR>CD8-PARP-Venus* eye discs treated with DTT for 1 hour exhibited a robust cleaved PARP signal, indicative of caspase activation. In eye discs treated with DTT for 2 to 3.5 hours, the PARP signal was stronger, demonstrating that the extent of caspase activation is proportional to the duration of ER stress. In both regimens, the PARP signal was significantly reduced with *derlin-1* knockdown (Figure 5A and 5B).

To further validate the pro-apoptotic function of Derlin-1 and to avoid potential off-target effect from *derlin-1* RNAi, we examined caspase activation and performed TUNEL (terminal deoxynucleotidyl transferase-dUTP nick end labeling) analysis in *derlin-1^null^* eye discs treated with ER stressor. While wild-type eye discs treated with DTT for two hours displayed extensive signals of cleaved caspase-3 and TUNEL staining, both apoptotic readouts were reduced in *derlin-1^null^* eye discs (Figure 5C, 5D and Figure S5). Collectively, these results demonstrate a role for Derlin-1 in ER stress-induced caspase activation.

To test whether Derlin-1 overexpression stimulates apoptotic pathway, we expressed Derlin-1 from *GMR-GAL4* (*GMR>derlin-1*) and used *UAS-CD8-PARP-Venus* to detect the extent of effector caspase activation. At larval stage, *GMR>LacZ* control and *GMR>derlin-1* eye discs showed mostly membrane-bound Venus and few processed anti-PARP staining, likely associate with apoptotic events in normal developmental eye. At the subsequent stages, while *GMR>LacZ* exhibited no detectable level of cleaved PARP, *GMR>derlin-1* pupal and adult eyes contained robust anti-PARP staining (Figure 5E). Similarly, while few cleaved caspase-3 signals were seen in *GMR>LacZ*, accumulated caspase-3 signals were readily observed in pupal and adult *GMR>derlin-1* retinas (Figure 5F). These results indicate that persistent Derlin-1 expression induces caspase activation.

### Counterbalance between Derlin-1 and TER94 is essential to prevent cytotoxicity

**Figure 6:**
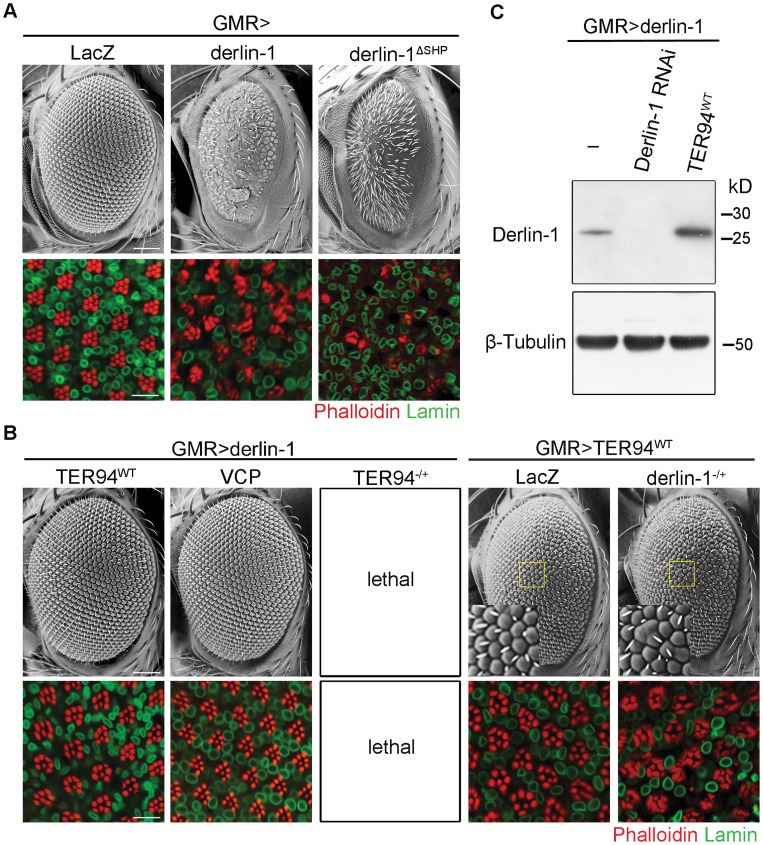
TER94 overexpression suppresses Derlin-1-associated cytotoxicity. (A and B) SEM (upper row) and confocal micrographs (lower row) of 1-day-old adult eyes with indicated transgenes expressed by *GMR-GAL4*. In all confocal panels, whole mount retinas are labeled with phalloidin (red) and anti-Lamin (green). (A) Eye-specific overexpression of Derlin-1 or SHP box deleted Derlin-1 causes a rough eye and photoreceptor disorganization (compared to the LacZ control). (B) Overexpression of either TER94 or human VCP suppresses Derlin-1-induced eye phenotypes (*GMR>derlin-1*), whereas reduction of TER94 (heterozygous for loss-of-function mutation; *TER94^−/+^*) in *GMR>derlin-1* results in lethality. A reciprocal genetic experiment shows mild phenotype in wild-type TER94 expressing eyes (*GMR>TER94^WT^*) is enhanced by reducing a copy of derlin-1 (heterozygous for *derlin-1^null^*; *derlin-1^−/+^*). Insets in SEM panels show enlarged views of the areas outlined in yellow. Fused ommatidia are evident in *GMR>TER94^WT^, derlin-1^−/+^*. Scale bars: 100 µm (SEM), 10 µm (confocal). (C) TER94 overexpression does not reduce Derlin-1 protein level. Western analysis of head lysates from *GMR>derlin-1* adults carrying indicated transgenes was probed with anti-Derlin-1 antibodies. The β-Tubulin bands serve as loading control.

Although increasing wild-type Derlin-1 rescued the rough eye phenotypes associated with pathogenic TER94 mutants, *GMR>derlin-1* alone, consistent with the result that excess Derlin-1 activates caspase, caused severe eye defects (Figure 6A). The toxic effect of Derlin-1 overexpression is not restricted to the eye development, as ubiquitous Derlin-1 overexpression by *hs-GAL4* or *act5C-GAL4* (*actin* promoter-directed GAL4) caused lethality at the mid-pupal stage. Interestingly, *GMR>derlin-1* phenotypes were suppressed by co-expressing *TER94^WT^* (Figure 6B) and more severe eye phenotypes, including reduced eye size and loss of photoreceptors, could be observed in *derlin-1^ΔSHP^* (Figure 6A), suggesting the binding to TER94 affects Derlin-1-induced dominant phenotype. Western analysis showed that the suppression was not due to a reduction in Derlin-1 level upon TER94 co-expression (Figure 6C). Co-expression of TER94^A229E^ also suppressed *GMR>derlin-1*, suggesting that the pathogenic mutation does not interfere with this TER94/derlin-1 interaction (Figure 1B). Moreover, overexpressing human VCP suppressed *GMR>derlin-1* (Figure 6B), suggesting the interaction between these two proteins is evolutionarily conserved.

The reciprocal suppression of Derlin-1 and TER94 overexpression suggests that the levels of these proteins under physiological conditions need to be coordinated. Indeed, in sensitized backgrounds like *GMR>derlin-1* or *GMR>TER94^WT^*, exacerbating the imbalance in Derlin-1 and TER94 levels worsened their phenotypes. For instance, although *TER94* heterozygotes were viable, *TER94* heterozygotes in *GMR>derlin-1* background resulted in lethality (Figure 6B). Conversely, while *GMR>TER94^WT^* had no apparent effect by itself in terms of eye roughness and cell loss, eye defect was manifest when it was expressing in *derlin-1* heterozygous background (Figure 6B).

### Derlin-1 overexpression induces ER stress and mitochondrial apoptosis

**Figure 7:**
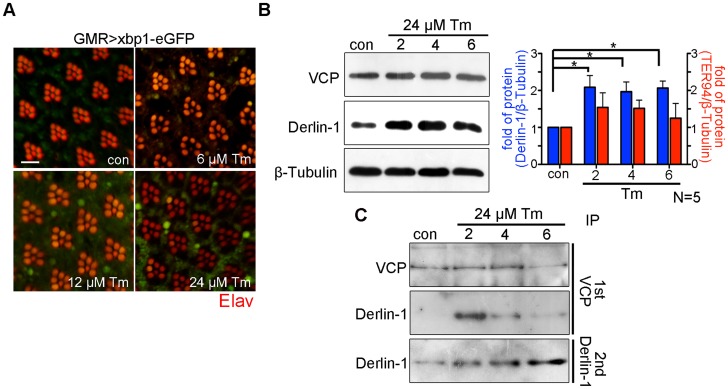
Prolonged ER stress increases Derlin-1 proteins that are not bound to TER94. (A) Confocal micrographs of retinas from *GMR>xbp1-eGFP* flies fed with different concentrations of Tunicamycin (Tm). The ER stress response to Tm treatment is dose-dependent, with 24 µM Tm eliciting robust Xbp1-eGFP signals. (B) Western analyses of lysates from flies fed with 24 µM Tm for 2, 4, and 6 days. The levels of endogenous Derlin-1 and TER94 (revealed by anti-Derlin-1 and anti-VCP antibodies) in response to continuous Tm treatment are compared to those from untreated (con). The β-Tubulin level is included as loading control. In the bar graph, endogenous Derlin-1 and TER94 levels (as shown in the Western in B) are normalized to loading controls and presented in fold change as compared to untreated control. Values shown represent mean ± SE from five independent experiments. *p<0.05; **p<0.01 (one-way ANOVA with Bonferroni's multiple comparison test). (C) Sequential IP (1st IP by anti-VCP and 2nd IP by anti-Derlin-1) of lysates from flies fed with 24 µM Tm for 2, 4, and 6 days. The immunoprecipitates from control and Tm-treated flies are probed with anti-VCP and anti-Derlin-1.

The cytotoxicity caused by Derlin-1 overexpression may mimic a situation, in which prolonged ER stress induces Derlin-1 expression to an extent that exceeds the level of cytoplasmic TER94. In that scenario, the level of free Derlin-1 (not associated with TER94) would increase as ER stress persists. To demonstrate this, sequential IP (first by VCP antibody and then by Derlin-1 antibody) was performed on adult flies treated with 24 µM tunicamycin (Tm), which has been used to cause ER stress for long duration (Figure 7A) [43]. Under this regimen, Tm-treated flies exhibited a high fatality rate at day 6, and most of them died by day 9. Although Derlin-1 level was elevated throughout the duration of Tm treatment (Figure 7B), the level of Derlin-1 associated with TER94 peaked at day 2 and significantly decreased afterward (Figure 7C). This analysis showed that free Derlin-1 level increased as ER stress persisted (Figure 7C). Together, these correlations support the idea that unbound Derlin-1 causes apoptosis.

**Figure 8:**
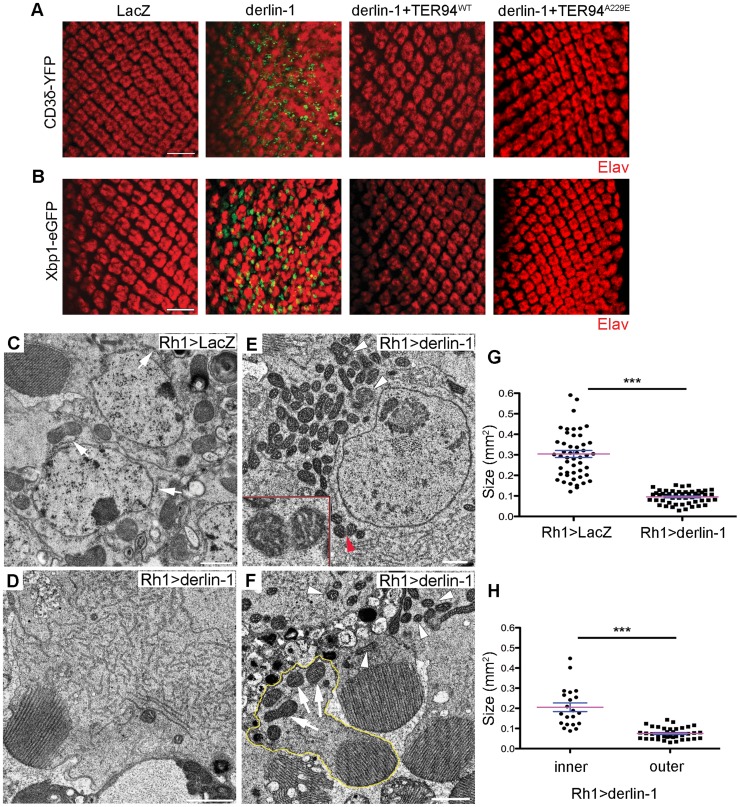
Derlin-1 overexpression impairs ER homeostasis and produces mitochondrial abnormality. (A and B) Confocal images of larval eye discs expressing CD3δ-YFP (A) and Xbp1-eGFP (B) probes (green), stained with anti-Elav antibodies (red) to label neuronal nuclei. The genotypes of eye discs include *GMR>LacZ* (control), *GMR>derlin-1* (*derlin-1* overexpression), *GMR>derlin-1>TER94^WT^* (overexpression of both *derlin-1* and *TER94*), and *GMR>derlin-1>TER94^A229E^* (overexpression of *derlin-1* and *TER94^A229E^*). (C–F) TEM micrographs of 18-day-old *Rh1>LacZ* control (C) and *Rh1>derlin-1* eyes (D–F). Unlike *Rh1>LacZ* (C), Derlin-1-overexpressing photoreceptors (D) contain an elevated level of ER-resembling tubular membranes. (E) Another TEM section shows that *Rh1>derlin-1* outer photoreceptors contain excessive ER membrane, as well as abnormal mitochondria with intracristal swelling (white arrowheads) and discontinuous membrane (red arrowhead). Inset shows higher magnification of mitochondrion pointed by red arrowhead. (F) A representative TEM micrograph shows that Derlin-1-overexpressing photoreceptors contain smaller mitochondria (white arrowheads). As *Rh1* promoter is active only in the outer photoreceptors, the mitochondria (arrows) in the inner photoreceptor (outlined in yellow) serve as an internal control. Scale bars: 10 µm (confocal). 1 µm (TEM). (G and H) Scatter dot plots of individual mitochondria in photoreceptor cells from four ultrathin sections. Mitochondrial size in outer photoreceptor cells of *Rh1>LacZ* and *Rh1>derlin-1* (G, *n* = 50 mitochondria per genotype), and in both inner and outer photoreceptor cells of *Rh1>derlin-1* (H, *n* = 21 and 37 mitochondria for inner and outer cells, respectively) were manually outlined to measure the size by ImageJ. Magenta bar and blue line represent mean ± SE in each group. ***p<0.001 (unpaired Student's t-test).

Excessive Derlin-1, because of its inability to interact with TER94, may interfere with the retrotranslocation of ERAD substrates. To test this, we examined the ERAD and UPR reporters in *GMR>derlin-1*. A robust CD3δ-YFP signal was seen in *GMR>derlin-1* eye discs, although the pattern differed from those seen in *derlin-1^null^*, as the ERAD reporter localized mainly to solid or ring-shaped puncta (Figure 8A). This abnormal CD3δ-YFP pattern suggests that Derlin-1 overexpression perturbs both the ERAD and the ER morphology. In addition, strong Xbp1-eGFP signals were detected in *GMR>derlin-1* eye discs, indicative of UPR activation (Figure 8B). Like other *GMR>derlin-1* phenotypes, the aberrant CD3δ-YFP accumulation and the Xbp1-eGFP signal were suppressed by TER94^WT^ or TER94^A229E^ overexpression (Figure 8A and 8B).

As the abnormal CD3δ-YFP pattern suggests a defect in the ER structures, cells overexpressing Derlin-1 from *Rh1>derlin-1* (*Rh1-GAL4;UAS-Derlin-1*) eyes were analyzed by TEM. *Rh1-GAL4*, active in the outer photoreceptors, was chosen for this analysis because in the same specimen, the structure of inner photoreceptor (where *Rh1* promoter is not active) served as the internal control. As shown in Figure 8, the cytosol of *Rh1>derlin-1* outer photoreceptors was often filled with ER-resembling tubular membranes (compare Figure 8C to 8D). This apparent ER expansion is consistent with the structural modification in cells under ER stress [44]. In addition, mitochondria with less than half of size as compared with *Rh1>LacZ* control were readily seen in *Rh1>derlin-1* outer photoreceptors (compare Figure 8C to 8E, and quantified in 8G). Some of these atypical mitochondria showed poor membrane integrity (Figure 8E, red arrowhead) and intracristal swelling (Figure 8E, white arrowheads), suggestive of apoptotic events [45]. The mitochondria in the inner photoreceptors appeared normal (Figure 8F, outlined in yellow; quantified in Figure 8H), indicating that the effect of Derlin-1 overexpression on mitochondrial morphology is cell–autonomous.

**Figure 9:**
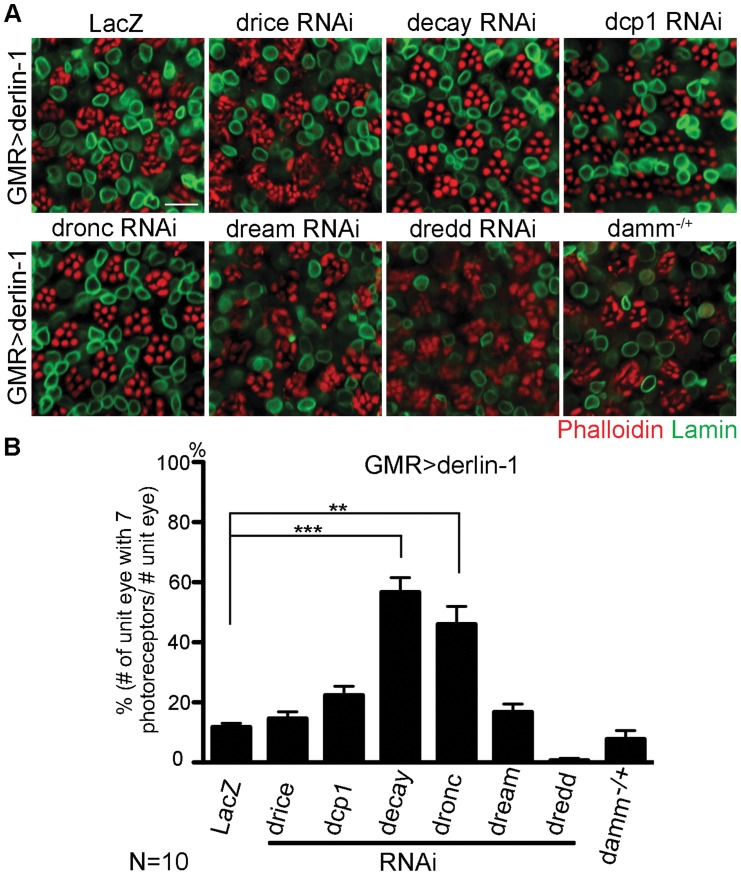
Derin-1 overexpression elicits canonical mitochondrial apoptosis. (A) Confocal images of 1-day-old *GMR>derlin-1* adult eyes co-expressing RNAi constructs of various caspases or in heterozygous *damm* background. Whole-mount adult eyes are stained with phalloidin (red) and anti-Lamin (green) to mark photoreceptor rhabdomeres and nuclear envelopes, respectively. Scale bar: 10 µm. (B) Quantification of the percentage of ommatidia (10 eyes for each genotype) containing normal complement of photoreceptors as shown in (A). Values shown represent mean ± SE. **p<0.01; ***p<0.001 (Student's t-test).

As excessive Derlin-1 induces mitochondrial abnormality, the cytotoxicity associated with Derlin-1 overexpression may be caused by damaged mitochondria to activate the canonical apoptotic pathway [46]. To confirm that Derlin-1-induced cytotoxicity requires caspase activation, we knocked down seven caspases in fly genome individually and tested their ability to alter the severity of *GMR>derlin-1* eye degeneration. While reduction of *drice*, *dcp1*, *dream*, *dredd*, and *damm* by RNAi lines or loss-of-function alleles showed no significant interaction, knockdown of *decay* and *dronc*, orthologs of effector caspase and caspase-9 respectively, suppressed the photoreceptor degeneration of *GMR>derlin-1* (Figure 9A and 9B). Since the activation of caspase-9 and the downstream effector caspase link to mitochondrial disruption, these data support that Derlin-1 overexpression activates intrinsic mitochondrial apoptosis.

### A putative α-helical domain mediates Derlin-1 cytotoxicity but is dispensable for suppressing TER94 disease mutant

**Figure 10:**
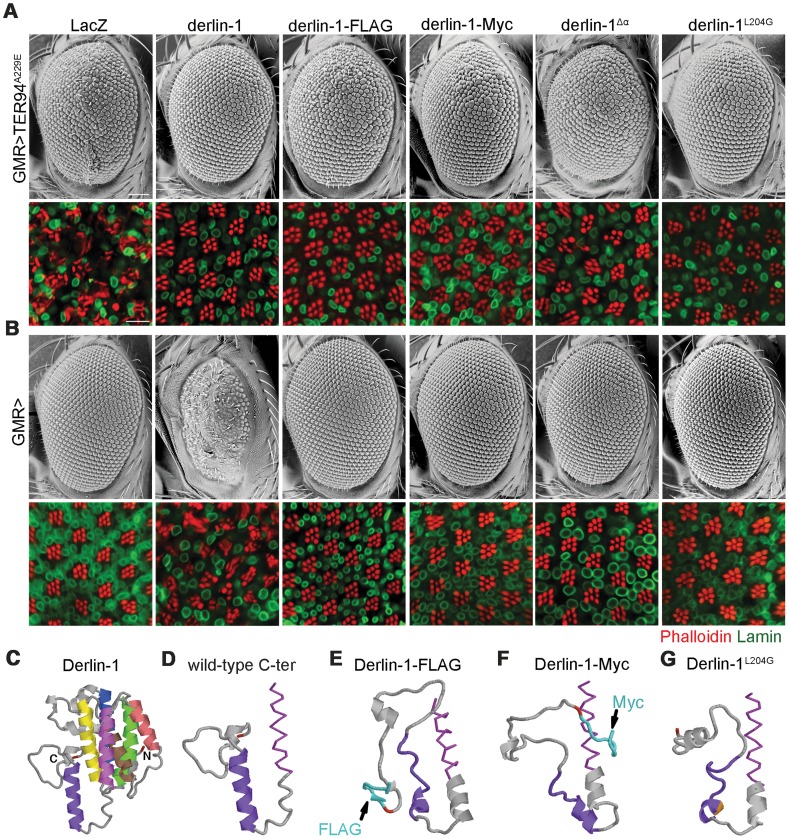
The C-terminal α-domain is required for Derlin-1 overexpression-induced cytotoxicity. (A and B) SEM (upper row) and confocal section (lower row) of 1-day-old adult fly eyes expressing the indicated transgenes with (A) or without (B) *TER94^A229E^* using *GMR-GAL4* driver. Phalloidin (red) and anti-Lamin antibodies (green) are used to label the rhabdomeres and the nuclear envelopes, respectively. Scale bars: 100 µm (SEM), 10 µm (confocal). (C–G) Structural prediction of Derlin-1 constructs by I-TASSER. (C) Full-length Derlin-1 features six major helixes (colored in blue, green, yellow, brown, red, and magenta from first to sixth helix), corresponding to the transmembrane domain. The C-terminal cytoplasmic tail contains the seventh helix (colored in purple). (D–G) The predicted sixth (magenta) transmembrane helix (shown as sticks view) and the C-terminal cytoplasmic tail (shown as cartoon view) of wild-type Derlin-1 (D), FLAG-tagged Derlin-1 (E), Myc-tagged Derlin-1 (F), and Derlin-1^L204G^ (G). The last residue of Derlin-1 is marked in red. Epitope tags and altered residue are marked in cyan (E and F) and gold (G), respectively.

Before the Derlin-1 antibody was available, we made transgenic flies carrying C-terminally FLAG-tagged Derlin-1 (*UAS-derlin-1-FLAG*) to monitor its expression and localization. This FLAG-tagged *derlin-1* rescued *derlin-1^null^* lethality and suppressed *GMR>TER94^A229E^* eye phenotype (Figure 10A), demonstrating that the chimera is functional. However, unlike *GMR>derlin-1*, a comparable level of Derlin-1-FLAG did not cause abnormal eye morphology (Figure 10B and Figure S6A). Similar results were obtained with Derlin-1-Myc (C-terminally Myc-tagged Derlin-1), albeit at a lower expression level (Figure S6A), indicating that this effect is not epitope specific (Figure 10A and 10B). Thus, it appears that the addition of epitope at the C-terminus of Derlin-1 abolishes its overexpression-dependent cytotoxicity. Structural modeling [47] suggested that all these Derlin-1 constructs were predicted to contain six major α-helical segments, corresponding to the proposed transmembrane domains of human Derlin-1 [26] (Figure 10C). However, the untagged Derlin-1 was predicted to contain another α-helix near its C-terminus (compare Figure 10D to 10E and 10F; aa. 201–215, hereby referred to as the α-domain), and it is possible that appending epitopes at the C-terminus disrupts this α-domain.

To experimentally validate the significance of this putative α-domain, we generated *UAS-derlin-1^
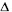
^α* (a truncation without the α-domain). As the SHP box remains intact in Derlin-1^
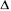
^α, expressing *derlin-1^
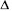
^α* still suppressed *GMR>TER94^A229E^*-induced eye phenotype (Figure 10A). However, unlike *GMR>derlin-1*, *GMR>derlin-1^
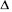
^α* eyes were normal and did not induce ER stress (Figure 10B and Figure S6B), supporting the notion that α-domain is critical for Derlin-1-mediated toxicity.

To avoid the complication that this C-terminal deletion broadly disrupts Derlin-1 conformation, we used structure prediction to look for residues that might be crucial for α-domain integrity. Substitution of leucine-204 by glycine (Derlin-1^L204G^) was predicted to disrupt α-domain (Figure 10G). Western analysis showed that the level of Derlin-1 expressions of wild-type and L204G constructs were comparable (Figure S6A). Similar to *derlin-1^
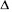
^α*, *derlin-1^L20^* ^4G^ suppressed *GMR>TER94^A229E^* eye phenotype (Figure 10A), but on its own showed normal eye morphology and no ER stress (Figure 10B and Figure S6B). Together, these data demonstrate that the C-terminal α-domain is not crucial for the suppression of pathogenic TER94, but required for Derlin-1-mediated cytotoxicity.

## Discussion

The genetic link between VCP missense mutations and a hereditary disorder, first discovered in IBMPFD, has recently been extended to other degenerative diseases, although the underlying pathogenic mechanism remains elusive. Here, we have identified Derlin-1 as a potent modifier of pathogenic TER94 mutant in a *Drosophila* IBMPFD model. Reduction and overexpression of Derlin-1 both exhibited genetic interactions with the disease-linked TER94 alleles, indicating that the disease pathogenesis is sensitive to Derlin-1 level. Our co-IP and pull-down assays demonstrated that Derlin-1 forms a complex with TER94 *in vivo*. The region required for this interaction is mapped to a putative SHP box near the C-terminal cytoplasmic tail. More importantly, we provided genetic evidence to show that the integrity of this SHP box, hence the ability to interact with TER94, is essential for the ability of Derlin-1 overexpression to suppress pathogenic TER94 mutants.

IBMPFD-linked alleles, like TER94^A229E^, retain ATPase activity and do not impair ERAD [13], [15], [36], implying that chronic ER stress is not the cause for this disease. We have shown that TER94^A229E^ depletes cellular ATP level and TER94^A229E^-associated photoreceptor degeneration can be suppressed by restoration of cellular ATP through genetic or environmental means [15]. Our observation that Derlin-1 overexpression restores the cellular ATP level, along with suppressing other TER94^A229E^-associated defects, further strengthens the notion that chronic ATP depletion results in IBMPFD-like symptoms. VCP/p97 carrying A232E mutation (analogous to TER94^A229E^) has been proposed to adopt a more mobile conformation with a higher ATPase activity [14]. It is possible that excessive Derlin-1 proteins in the ER (caused by the overexpression) keep TER94^A229E^ in a less mobile conformation, thereby negating the ATP-depleting effect of the disease mutant. Indeed, the ATPase activity of TER94^A229E^ is reduced in the presence of Derlin-1 overexpression. Collectively, these results suggest that excess Derlin-1 reduces TER94 ATPase activity by directly binding to TER94.

Alternatively, as VCP has been implicated in multiple processes, TER94 overexpression may cause cytotoxicity via a mechanism unrelated to ATP expenditure. TER94 overexpression, in the absence of Derlin-1 overexpression, generates excessive TER94 in the cytoplasm, which may titrate out a cytoplasmic factor critical for cell survival. Indeed, expression of pathogenic VCP in other models has been shown to cause cytosolic accumulation of autophagosome-associated proteins [17], [18] and TDP-43 [17], [19]. In this scenario, overexpressed Derlin-1 may negate this titration by restricting TER94 to the ER, thereby suppressing TER94-associated phenotypes. In support of this, the Derlin-1 deletion that cannot localize to the ER fails to suppress *GMR>TER94^A229E^*.

Consistent with the notion that Derlin-1 acts in the retrotranslocation [21], [22], [26], [48]–[51], misfolded proteins accumulate in the ER in *derlin-1^null^* mutants, indicative of disrupted ERAD. The fact that loss of Derlin-1 function in both fly and mouse [52] results in lethality suggests that the maintenance of ER homeostasis is critical for animal survival. Derlin-1 expression is elevated in cells treated with DTT, Tm, or cold temperature, suggesting that Derlin-1 is a part of the adaptive program to alleviate ER stress. This response is evolutionarily conserved, as chemical-induced ER stress in yeast also elevates *DER1* level [53]. Interestingly, cold treatment does not increase TER94 expression, although the association of TER94 with the ER appears elevated in a Derlin-1-dependent manner. These observations suggest that in the absence of ER stress, Derlin-1 and its associated retrotranslocation machinery (i.e. TER94/Derlin-1 complex) is limited. In response to ER stress, additional Derlin-1 is synthesized and complexes with existent TER94 to restore ER homeostasis.

While the function of VCP in ERAD pathway is firmly established, it is unclear how this versatile AAA ATPase is recruited to the ER to process misfolded substrates [35]. The observation that the association of TER94 with the ER is Derlin-1-dependent suggests that Derlin-1 acts as a recruiting factor. This notion is consistent with the fact that mammalian VCP binding to Derlin-1 is required for efficient dislocation of ERAD substrates [26]. As VCP has multiple functions, a tight control of the recruiting co-factor expression could ensure the ATPase is utilized efficiently.

Given the importance of Derlin-1 in ERAD and its upregulation upon ER stress, why does overexpression of Derlin-1, a component of the machinery responsible for restoring ER homeostasis, cause ER stress and activate pro-apoptotic signals? *GMR>derlin-1* defects are suppressed by co-expressing *TER94* and exacerbated by reduction of TER94 function, suggesting that it is not the Derlin-1 overexpression per se, but the imbalance between Derlin-1 and TER94 levels that causes ER stress and cell death. As ER stress induces Derlin-1 expression, but not TER94, it is possible that Derlin-1 has another function in addition to maintaining ER homeostasis, depending of the availability of cytoplasmic TER94. In the event of moderate ER stress, the level of cytoplasmic TER94 remains in excess and Derlin-1 induced by low level of ER stress would recruit TER94 to the ER to retrotranslocate misfolded proteins. In the event of severe or chronic ER stress, induced Derlin-1 expression may exceed TER94 level, which would result in a population of Derlin-1 not bound to TER94. Unlike the Derlin-1/TER94 complexes that act to restore ER homeostasis, the unbound Derlin-1 would worsen the ER stress, ensuring the initiation of pro-apoptotic signals. As ER stress-induced UPR needs to juggle between the cyto-protective and pro-apoptotic functions in response to ER stress, the level of unbound Derlin-1 may act as a sensor of cells with irreparable ER stress. In support of this, we show that while Tm treatment initially enhances Derlin-1/TER94 association, the level of unbound Derlin-1 increases in cells with prolonged ER stress. Furthermore, this hypothesis predicts that overexpression of a Derlin-1 mutant incapable of binding to TER94 should enhance the cytotoxicity, which is exactly what we observed with *GMR>derlin-1^
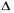
SHP^*. While it is unclear whether the proposed mechanism exists in other systems, a genome-wide expression analysis in yeast has revealed that UPR up-regulates Der1, but not CDC48 [53].

Although the exact mechanism of how excessive Derlin-1 induces UPR and apoptosis remains enigmatic, we provide evidence that it relies on a novel C-terminal motif. It may be that this α-domain facilitates the interaction of Derlin-1 with another retrotranslocation factor, and this complex acts as a dominant negative without TER94 bound. Alternatively, this α-domain may have an active role in disrupting ERAD, and the binding of TER94 to the SHP motif (adjacent to the α-domain) prevents this α-domain from being exposed. Identifying factors interacting with this α-domain will be needed to define the mechanism for this Derlin-1 overexpression-dependent cytotoxicity.

## Materials and Methods

### *Drosophila* genetics and molecular biology

Flies were raised on standard cornmeal food at 22°C in 12 hrs light/dark cycles unless otherwise noted. The *derlin-1^l(2)SH1964^*, *UAS-Lys-GFP-KDEL* [54] and *UAS-tub-GAL80^ts^*, and all transgenic RNAi lines were obtained from Szeged *Drosophila* Stock Center (Hungary), Bloomington Stock Center (Indiana, USA), or Vienna *Drosophila* RNAi Center (Austria), respectively. *UAS-xbp1-eGFP* and *UAS-CD8-PARP-Venus* were generous gifts from Drs. Hermann Steller and Darren Williams. *UAS-CD3δ-YFP*, *GMR-GAL4*, *Rh1-GAL4*, *hs-GAL4*, and *damm^f022091^* flies have been previously described [15], [55].

To construct pUAST-derlin-1, pUAST-sip3, and pUAST-ufd1, *Drosophila* derlin-1 (GH08782), Sip3 cDNA (GH11117), and Ufd1 (GH18603) cDNA clones were obtained from *Drosophila* Genomics Resource Center (Indiana, USA), and subcloned into pUAST as EcoRI-XhoI fragments. To construct pUAST-GFP-Derlin-1-CT, Derlin-1 cytoplasmic tail (corresponding to aa. 189–245) was PCR amplified (F, 5′GAGCGGCCGCTAATTCGCAGGA3′; R, 5′GCGCTCGAGTTCAGTTGCGACCC3′) and cloned into pUAST-GFP as a NotI-XhoI fragment, resulting in an in-frame fusion to the C-terminus of GFP. To generate tagged derlin-1 constructs, FLAG or Myc sequences were appended in frame to the C-terminus of derlin-1 by PCR. Derlin-1 deletion and point mutation variants were generated by PCR or site-directed mutagenesis (QuikChange, Stratagene). For pUAST-derlin-2 construct, a set of primers were designed (see Table S2) to amplify the full-length derlin-2 cDNA by RT-PCR (below) of wild-type fly RNA, and subsequently cloned into pUAST as EcoRI-XhoI fragments. For pUAS-caspase RNAi constructs, 22 nt sequences corresponding to mature Mir 6.1 were replaced with sequences perfectly complementary to the coding sequences of the caspase [56]. The targeted sites are listed in Table S2. All constructs were verified by sequencing, and transgenic flies were generated by P element-mediated transformation.

For RT-PCR, 2 µg of total RNA, isolated with the TRIzol reagent (Invitrogen), was used for reverse transcription (SuperScript II, Invitrogen). Primer sets and conditions for subsequent PCR amplification are listed in Table S2.

### Antibody production and immunohistochemistry

To generate polyclonal anti-Derlin-1 antibody, a peptide (N-SRAPPRQATESPWG-C) corresponding to the C-terminus of Derlin-1 was synthesized and used for immunization (GenedireX, Taiwan). In Western analysis, this antibody detected a prominent ∼27 kD band in control extract, correlating well with the predicted molecular mass of Derlin-1 protein (28.25 kD). This antibody is specific, as the intensity of this 27 kD band was elevated in *hs>derlin-1* and absent in *derlin-1^l(2)SH1964^* extracts.

Whole-mount preparation of fly eyes was performed as previously described [57]. The primary antibodies used were anti-Derlin-1 (1∶50), anti-VCP (1∶50, GeneTex, Taiwan), anti-Elav (1∶50, Developmental Studies Hybridoma Bank, DSHB), anti-LaminDm (1∶20, DSHB), anti-cleaved PARP (1∶50, abcam), and anti-cleaved caspase-3 (1∶50, Cell Signaling). For TUNEL staining, eyes were dissected in PBS and then treated with fresh prepared DTT. Samples were fixed and permeabilized according to the manufacture's instruction (ApopTag Red, Millipore). Fluorescently conjugated secondary antibodies (Jackson ImmunoResearch Laboratories) were used at 1∶100 dilutions. Rhodamine-conjugated phalloidin (Sigma) were used (10 µM) to label F-Actin-enriched rhabdomeres. All fluorescent images were collected on Zeiss LSM-510 or 710 confocal microscopes, and processed with Photoshop CS. To compare fluorescent-labeled signals, samples of different genotypes were prepared and imaged with identical procedures and confocal settings.

For quantifying co-localization with KDEL-eGFP, Pearson's co-localization coefficient (PCC) analyses were performed using the NIH ImageJ Just Another Colocalization Plugin (JACoP). JACoP directly provides the PCC values for a set of images in the red and green channels, ranging from 1 for perfectly correlation to -1 for inversely correlation (a PCC value of 0 indicates no correlation between the two channels). The provided PCC values are independent of the fluorescence intensity.

### GST pull-down assay

For GST pull-down assays, various Derlin-1 C-terminal regions were amplified by PCR, and subcloned into the pGEX-6p3 vector as EcoRI-XhoI fragments. To generate His-TER94 constructs, TER94 fragments were amplified by PCR, and subcloned into pET-28a(+) using EcoRI-XhoI sites. Sequences of the primers used can be found in Table S2.

To produce GST- and His-fusion proteins, BL21 cells carrying appropriate plasmids were grown at 37°C, induced with 0.1 mM isopropyl-β-D-thiogalactopyranoside (IPTG), pelleted, and resuspended in protein extraction buffer (1% CHAPS, 100 mM KCl, 0.1 M Tris-HCl (pH 7.5), 20 mM Hepes, 10 mM EDTA, 5% Glycerol) supplemented with protease inhibitor cocktail (Roche). The fusion proteins were purified with MagneGST or MagneHis Protein Purification System kits (Promega) according to manufacturer's instructions. For the pull-down, immobilized GST-fusion proteins were incubated with either purified His-TER94 or fly protein extracts at 4°C overnight. After washing, bound proteins were eluted and analyzed by Western blots.

### Immunoprecipitation and immunoblotting

For immunoprecipitations, transgenic flies driven by *hs-GAL4; tub-GAL80^ts^* were treated with three cycles of heat shock (one hour at 37°C followed by one hour at 25°C) to induce transgene expressions. Whole fly protein lysates were collected. Anti-Derlin-1 antibody was bound to protein A-agarose beads (Sigma) by incubation for 4 hours at 4°C, followed by three washes with lysis buffer (0.5% Triton X-100, 10 mM Tris-HCl, 5 mM EDTA, 100 mM NaCl, pH 7.5). The antibody/agarose beads were then incubated with pre-cleared lysate at 4°C overnight, washed four times with lysis buffer, and eluted by Laemmli buffer for Western analysis.

For TER94 co-IP after the cold treatment, wild-type flies were kept at 0°C for two hours, allowed to recover for four hours at 25°C, and then subjected to lysate preparation. For sequential IP, protein lysates from flies fed with 24 µM tunicamycin were subjected to one round of IP using anti-VCP-bound protein A-agarose beads. After incubation for 4 hours at 4°C and centrifugation, the unbound supernatants were subjected to a second round of IP using anti-Derlin-1-bound protein A-agarose beads. Anti-VCP antibody (GeneTex, Taiwan) was used for immunoprecipitation. The primary antibodies were used at the following dilutions: anti-VCP (1∶1000, Cell Signaling), anti-β-Tubulin (1∶10000, DSHB), anti-Derlin-1 (1∶2000), anti-β-Actin (1∶5000, abcam), anti-6XHis (1∶3000, GeneTex), anti-Ufd1 (1∶10000, GeneTex), and anti-GST (1∶50000, Cell Signaling). HRP-conjugated secondary antibodies (Jackson ImmunoResearch Laboratories) were used at 1∶10000 dilutions. For immunoblotting after immunoprecipitation, the Clean-Blot IP detection kit (Thermo) was used. ImageJ was used to quantify the immunoblots.

### ATP level and in-gel ATPase activity assays

Cellular ATP measurements were performed as previously described [15]. ATPase activity analysis was performed as previously described with modifications [58]. Briefly, *hs-GAL4; tub-GAL80^ts^* flies were treated as above to induce transgene expressions. Flies were then homogenized in grinding buffer (50 mM NaCl, 50 mM Imidazole/HCl, 2 mM 6-aminohexanoic acid, 1 mM EDTA, 5% Glycerol, pH 7.0, supplemented with 1% digitonin and protease inhibitor (Roche)), and the lysates were centrifuged at 16,000xg for 10 min at 4°C to remove cell debris. 80 µg of proteins were loaded onto 7.5% clear-native PAGE with the addition of 0.1% Ponceau S to mark the sample front during electrophoresis (45 volts/30 min followed by 100 volts/20 min and then 250 volts/2 hrs at 4°C). The gels were then washed in 35 mM Tris, 270 mM Glycine, pH 8.3 for 2 hrs at 25°C and then incubated in ATPase assay buffer (35 mM Tris, 270 mM Glycine, 14 mM MgSO_4_, 0.2% Pb(NO_3_)_2_, 8 mM ATP, pH 8.3) at 25°C overnight. ATP hydrolysis caused lead phosphate to form white precipitates, which turned into brownish-black color after treatment with 1% ammonium sulfide. Quantification of band intensities in photographed gels was analyzed with ImageJ (NIH Image).

### Electron microscopy

Scanning electron micrographs of adult eyes were obtained with TM-1000 SEM (Hitachi) as previously described [15]. For transmission electron microscopy, samples and ultrathin sections were prepared as previously described [59], and imaged with HT7700 TEM (Hitachi).

## Supporting Information

Figure S1 Knockdown of endogenous Sip3 and Ufd1 levels with the expression of RNAi constructs. (A) RT-PCR and (B) Western blotting results show the knockdown efficacy of (A) *sip3* and (B) *ufd1* RNAi lines used in Figure 1. The normalized ratios of *sip3* or *ufd1* expressions to controls are indicated at the bottom of the gels.
(TIF)

Figure S2 The C-terminal cytosolic domain of fly Derlin-1, but not Derlin-2, binds to TER94. (A) A GST-Derlin-1-C-terminal fusion pulls down both wild type and A229E mutant of His-TER94 in vitro. The bound proteins are analyzed by Western blots with anti-6XHis and anti-GST antibodies. (B) GST-fusions containing the C-terminal cytosolic tails of Derlin-1 and Derlin-2 are used to pull down TER94^A229E^ from *GMR>TER94^A229E^* head lysate. The bound proteins are analyzed by Western blots with anti-VCP and anti-GST antibodies. The input blot represents 10% of the head lysate used for each pull-down experiment.
(TIF)

Figure S3 Cold shock treatment induces UPR response. Confocal images of *GMR>xbp1-eGFP* adult eyes kept at RT (–) or treated with a 2hr cold shock at 0°C. The retinas express xbp1-eGFP, an UPR probe, under the *GMR* control, and are stained with phalloidin (red) to outline rhabdomeres. The presence of GFP puncta (indicated by arrows) represents the activation of UPR. Scale bar: 10 µm.
(TIF)

Figure S4 Cold shock-induced ER stress does not enhance TER94 protein level. (A) Quantitative Western of endogenous TER94 proteins from flies subjected to 2 hrs cold shock at 0°C. Lysates from wild type flies (con; without cold treatment) and those recovered after the cold shock for the indicated time periods are probed with anti-VCP antibody. β-Tubulin serves as loading control. (B) Results from five independent quantitative Western experiments in (A) are shown. TER94 protein levels, after normalized to loading controls, are shown in fold change as compared to untreated control. Values shown represent mean ± SE. No significant difference after testing by one-way ANOVA.
(TIF)

Figure S5 Derlin-1 is critical for ER stress-induced apoptosis. (A) Confocal images of TUNEL-labeled (green) wild-type and *derlin-1^null^* eye discs co-stained by anti-Elav (red). Positive control (PC) eye disc was treated with DNase I, whereas the negative control (NC) did not include TdT (terminal deoxynucleotidyl transferase) in the reaction. The experimental eye discs (wild-type and *derlin-1^null^*) were treated with 5 mM DTT for 2 hrs. Scale bars: 10 µm. (B) Quantification of TUNEL puncta is shown in A. Punta numbers were normalized to the disc size. Values represent mean ± SE. ***p<0.001 (Student's t-test).
(TIF)

Figure S6 Disruption of Derlin-1 C-terminus negates the ability of Derlin-1 overexpression to impair ERAD and induce UPR. (A) Protein levels expressed from different Derlin-1 constructs, with the exception of Derlin-1-Myc, are comparable. Extracts of indicated derlin-1 transgenic constructs expressed from *GMR-GAL4* are analyzed by Western blot with anti-Derlin-1 antibody. For loading control, the blot was re-probed with anti-β-Tubulin antibody. (B) Confocal images of larval eye discs expressing indicated Derlin-1 constructs under *GMR* control are immunostained with anti-Elav antibody to label neuronal nuclei. The eye discs also express CD3δ-YFP or Xbp1-eGFP to monitor ERAD function and UPR response, respectively. Scale bar: 10 µm.
(TIF)

Table S1 The score list of knocked-down genes.
(DOCX)

Table S2 Primer sequence used in generating the indicated constructs and experiments.
(DOC)
