# Supplementary figures and images for: Derlin-1 Regulates Mutant VCP-Linked Pathogenesis and Endoplasmic Reticulum Stress-Induced Apoptosis
Source: PLoS Genet. 2014 Sep 25;10(9):e1004675. doi: 10.1371/journal.pgen.1004675 (PMC4177747; doi:10.1371/journal.pgen.1004675)

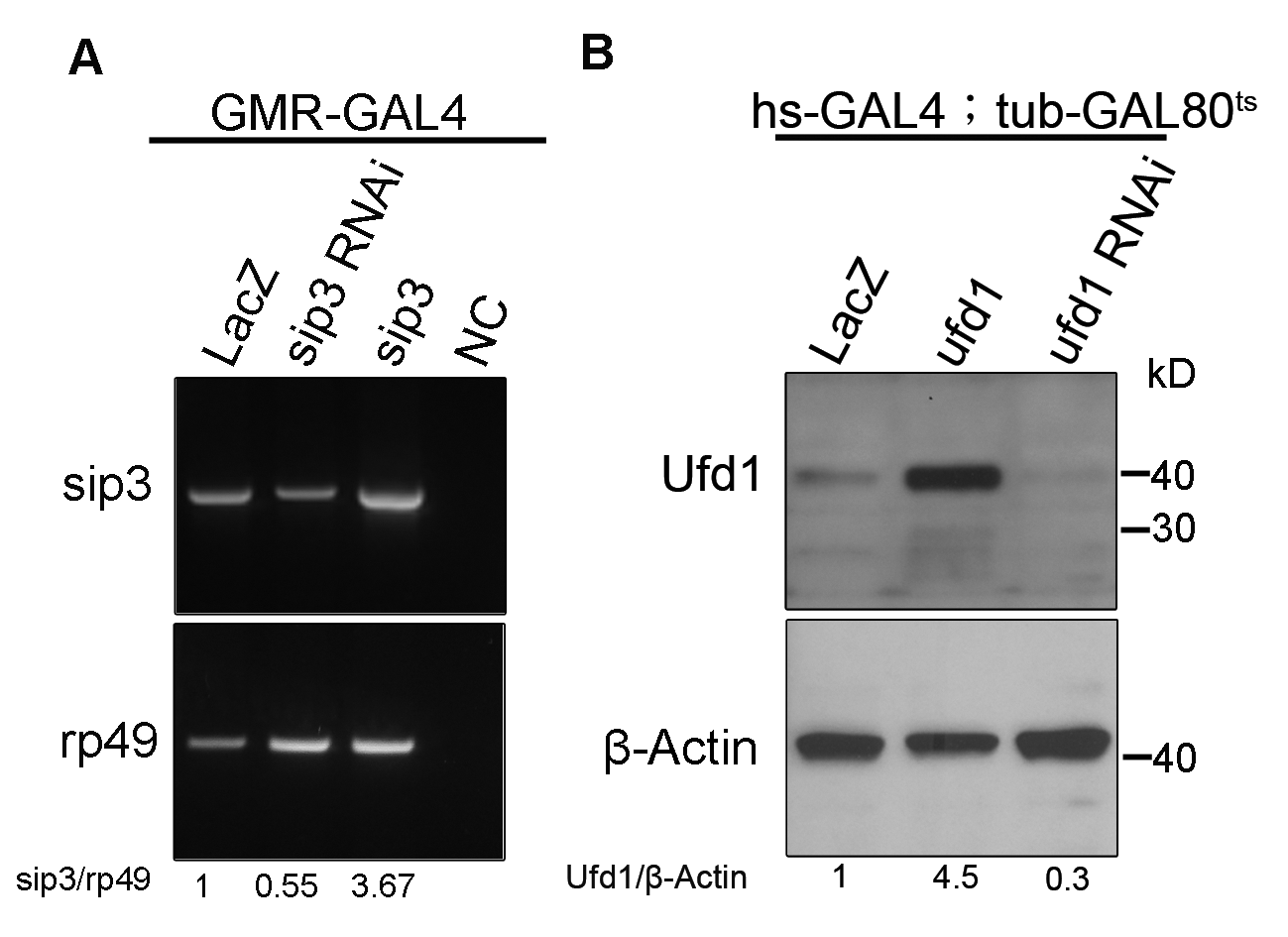

Supplement: Figure S1 — Knockdown of endogenous Sip3 and Ufd1 levels with the expression of RNAi constructs. (A) RT-PCR and (B) Western blotting results show the knockdown efficacy of (A) sip3 and (B) ufd1 RNAi lines used in Figure 1. The normalized ratios of sip3 or ufd1 expressions to controls are indicated at the bottom of the gels. (TIF) [file pgen.1004675.s001.tif]

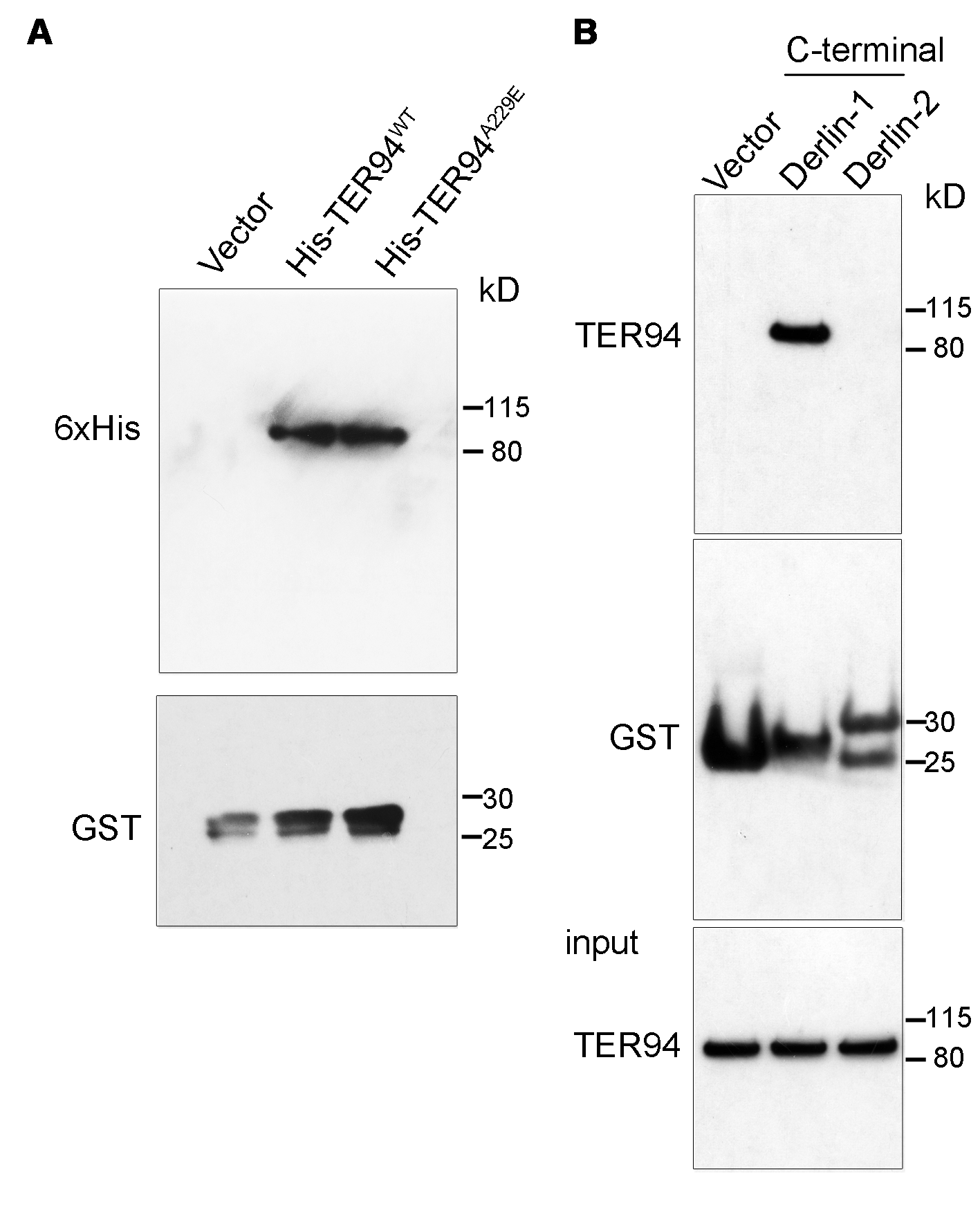

Supplement: Figure S2 — The C-terminal cytosolic domain of fly Derlin-1, but not Derlin-2, binds to TER94. (A) A GST-Derlin-1-C-terminal fusion pulls down both wild type and A229E mutant of His-TER94 in vitro. The bound proteins are analyzed by Western blots with anti-6XHis and anti-GST antibodies. (B) GST-fusions containing the C-terminal cytosolic tails of Derlin-1 and Derlin-2 are used to pull down TER94A229E from GMR>TER94A229E head lysate. The bound proteins are analyzed by Western blots with anti-VCP and anti-GST antibodies. The input blot represents 10% of the head lysate used for each pull-down experiment. (TIF) [file pgen.1004675.s002.tif]

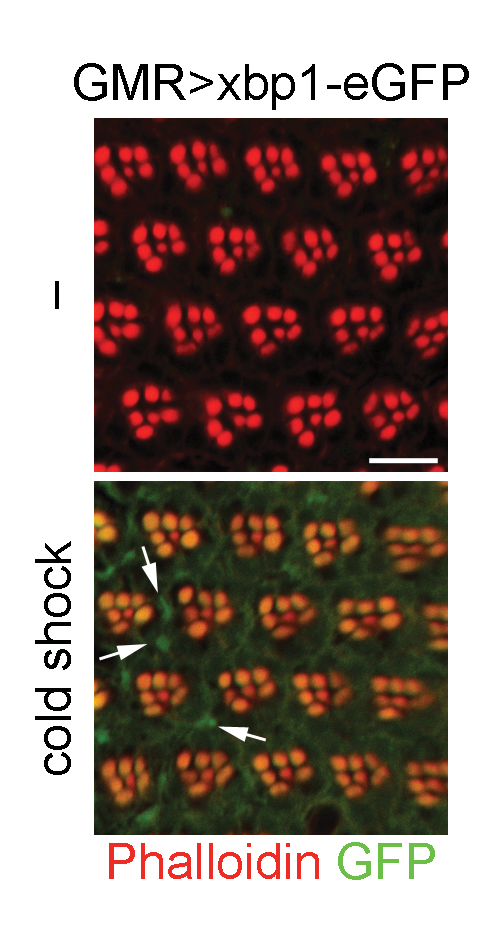

Supplement: Figure S3 — Cold shock treatment induces UPR response. Confocal images of GMR>xbp1-eGFP adult eyes kept at RT (–) or treated with a 2hr cold shock at 0°C. The retinas express xbp1-eGFP, an UPR probe, under the GMR control, and are stained with phalloidin (red) to outline rhabdomeres. The presence of GFP puncta (indicated by arrows) represents the activation of UPR. Scale bar: 10 µm. (TIF) [file pgen.1004675.s003.tif]

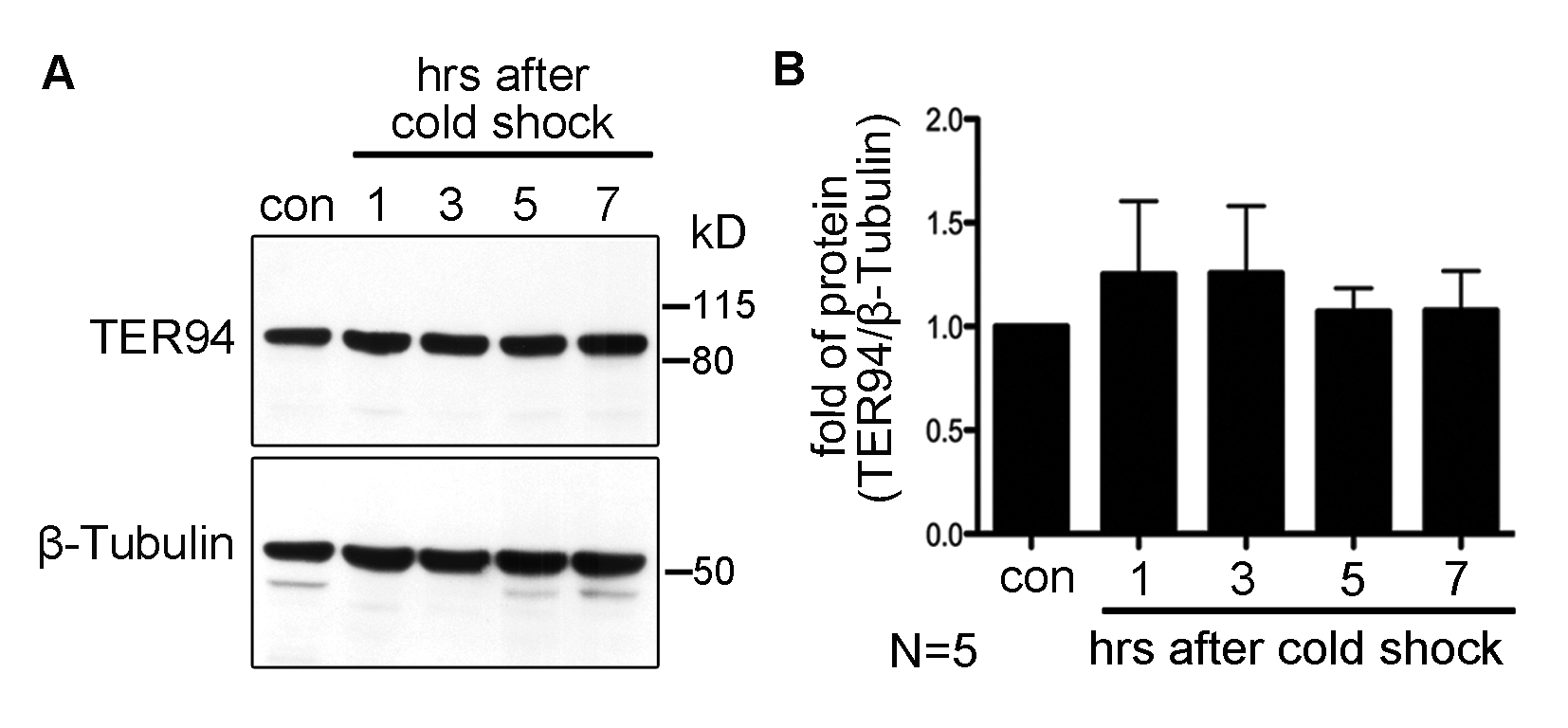

Supplement: Figure S4 — Cold shock-induced ER stress does not enhance TER94 protein level. (A) Quantitative Western of endogenous TER94 proteins from flies subjected to 2 hrs cold shock at 0°C. Lysates from wild type flies (con; without cold treatment) and those recovered after the cold shock for the indicated time periods are probed with anti-VCP antibody. β-Tubulin serves as loading control. (B) Results from five independent quantitative Western experiments in (A) are shown. TER94 protein levels, after normalized to loading controls, are shown in fold change as compared to untreated control. Values shown represent mean ± SE. No significant difference after testing by one-way ANOVA. (TIF) [file pgen.1004675.s004.tif]

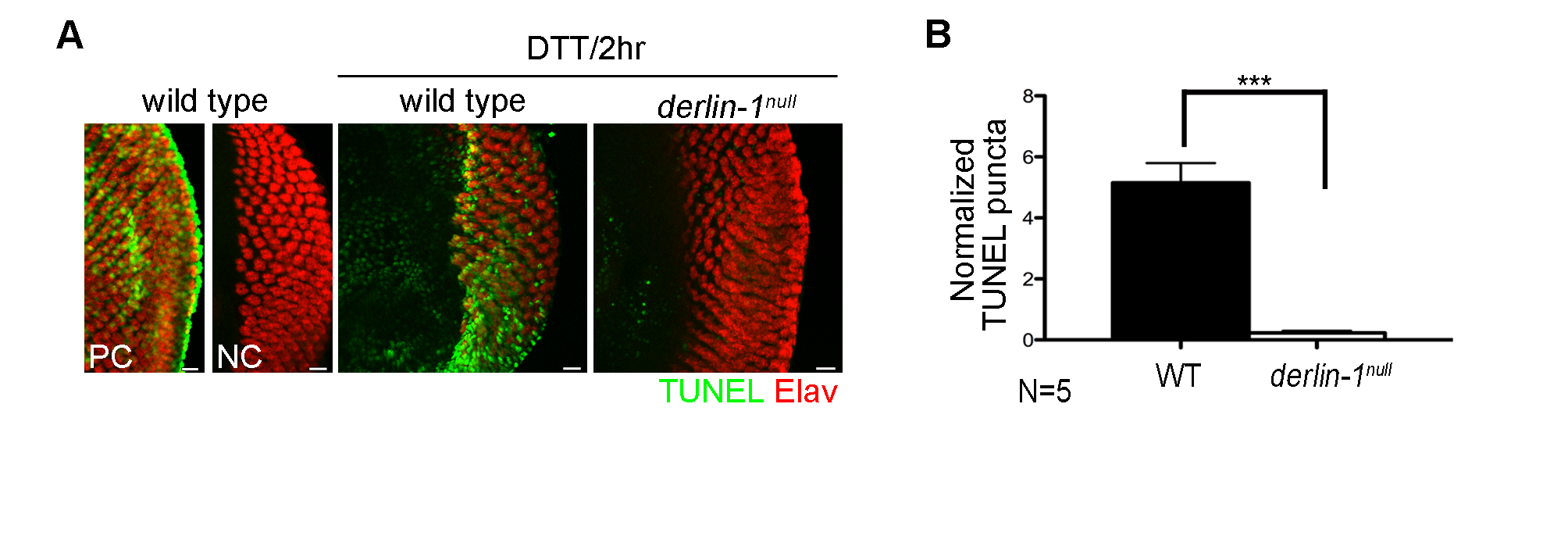

Supplement: Figure S5 — Derlin-1 is critical for ER stress-induced apoptosis. (A) Confocal images of TUNEL-labeled (green) wild-type and derlin-1null eye discs co-stained by anti-Elav (red). Positive control (PC) eye disc was treated with DNase I, whereas the negative control (NC) did not include TdT (terminal deoxynucleotidyl transferase) in the reaction. The experimental eye discs (wild-type and derlin-1null) were treated with 5 mM DTT for 2 hrs. Scale bars: 10 µm. (B) Quantification of TUNEL puncta is shown in A. Punta numbers were normalized to the disc size. Values represent mean ± SE. ***p<0.001 (Student's t-test). (TIF) [file pgen.1004675.s005.tif]

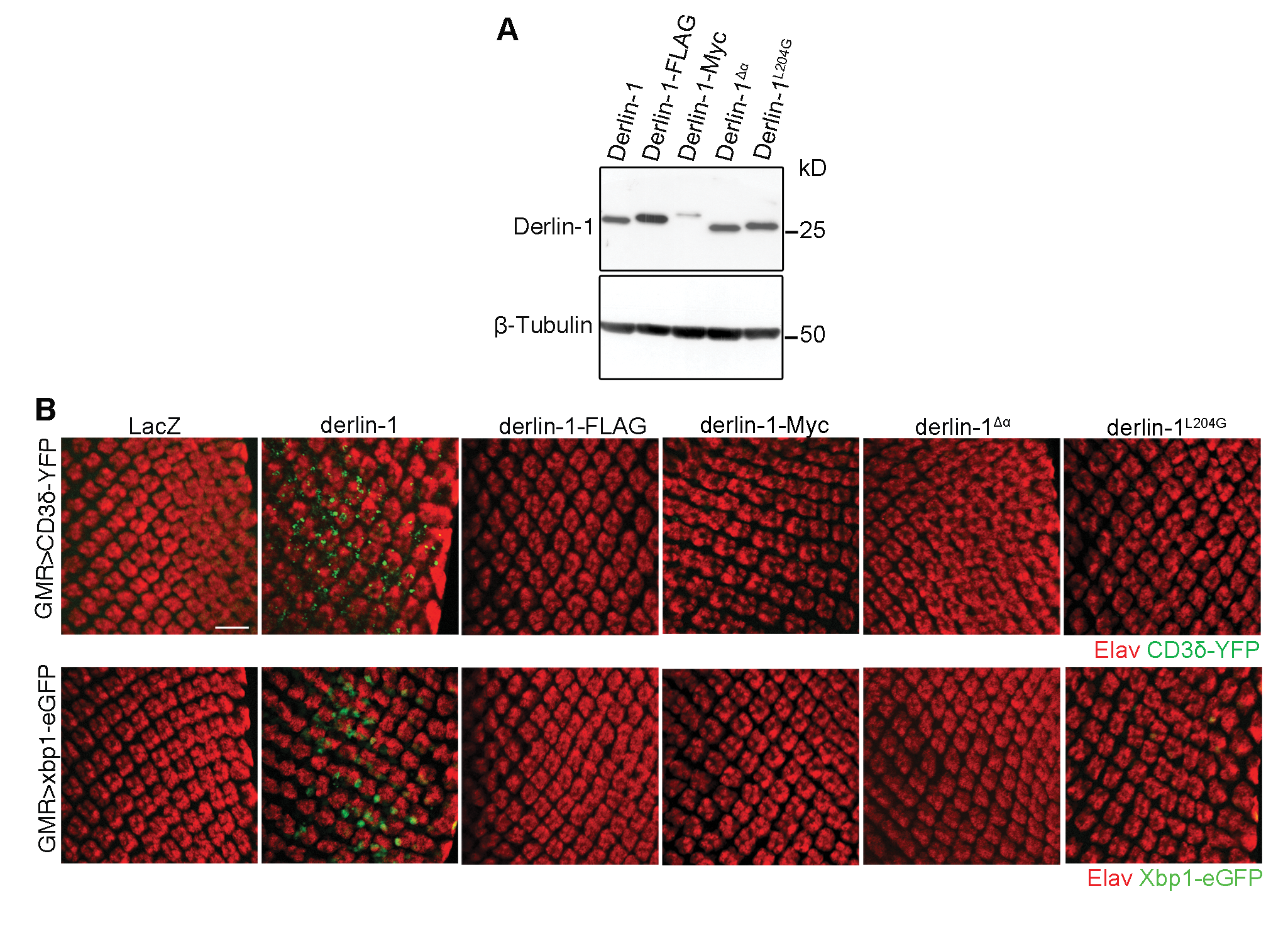

Supplement: Figure S6 — Disruption of Derlin-1 C-terminus negates the ability of Derlin-1 overexpression to impair ERAD and induce UPR. (A) Protein levels expressed from different Derlin-1 constructs, with the exception of Derlin-1-Myc, are comparable. Extracts of indicated derlin-1 transgenic constructs expressed from GMR-GAL4 are analyzed by Western blot with anti-Derlin-1 antibody. For loading control, the blot was re-probed with anti-β-Tubulin antibody. (B) Confocal images of larval eye discs expressing indicated Derlin-1 constructs under GMR control are immunostained with anti-Elav antibody to label neuronal nuclei. The eye discs also express CD3δ-YFP or Xbp1-eGFP to monitor ERAD function and UPR response, respectively. Scale bar: 10 µm. (TIF) [file pgen.1004675.s006.tif]
