# Supplementary material for: Derlin-1 Regulates Mutant VCP-Linked Pathogenesis and Endoplasmic Reticulum Stress-Induced Apoptosis
Source: PLoS Genet. 2014 Sep 25;10(9):e1004675. doi: 10.1371/journal.pgen.1004675 (PMC4177747; doi:10.1371/journal.pgen.1004675)
Supplement: Table S1 — The score list of knocked-down genes. (DOCX) [file pgen.1004675.s007.docx]

**
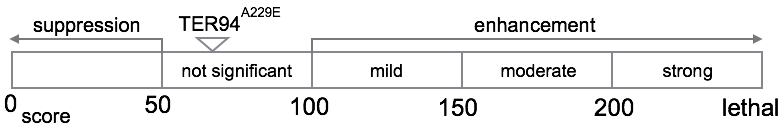
 Table S1**. The score list of knocked-down genes.

| Knocked-down genes | Score* | Modification |
| --- | --- | --- |
| *Cofactors* | | |
| Ufd1 | Lethal | Strong enhancement |
| Npl4 | 150-200 | Moderate enhancement |
| Eyc | 100-150 | Mild enhancement |
| Faf | 50-100 | Not significant |
| *Association proteins* | | |
| Sip3 | >200 | Strong enhancement |
| Svip | 150-200 | Moderate enhancement |
| Derlin-1 | >200 | Strong enhancement |
| Derlin-2 | 0-50 | Suppression |
| Hrd3 | 100-150 | Mild enhancement |
| TDP43 | 50-100 | Not significant |
| Usa1 | 0-50 | Suppression |

* Score refers to the roughness area that corresponds to the number of ommatidium in normal compound eyes. For *GMR>TER94^A229E^*, the areas range between 50 to 100 ommatidia. Two SEM micrographs were examined for each tested gene.
