# Supplementary material for: Derlin-1 Regulates Mutant VCP-Linked Pathogenesis and Endoplasmic Reticulum Stress-Induced Apoptosis
Source: PLoS Genet. 2014 Sep 25;10(9):e1004675. doi: 10.1371/journal.pgen.1004675 (PMC4177747; doi:10.1371/journal.pgen.1004675)
Supplement: Table S2 — Primer sequence used in generating the indicated constructs and experiments. (DOC) [file pgen.1004675.s008.doc]

**Primer sets used to generate wild type and modified Derlin-1 for subcloning to the pUAST plasmid**

| Construct | Sequence |
| --- | --- |
| derlin-1 | F, 5’ ACACGAGAATTCCACACAATGGAC 3’ |
|  | R, 5’ GTCTAGCGCTTCAGAGAGTTTCGAGT 3’ |
| derlin-1-FLAG | F, 5’ GGGAATTGGGAATTCGGCACG 3’ |
|  | R, 5’ GGCGATCTCGAGTCACTTGTCGTCATC TTGTAGTCTCCGTTGCGACCCAAGGTCATGC 3’ |
| derlin-1-Myc | F, 5’ GGGAATTGGGAATTCGGCACG 3’ |
|  | R, 5’ TCTCGAGCAGATCTTCTTCAGAAATAAGTTT  TTGTTCGTTGCGAC 3’ |
| derlin-1^∆SHP^ | F, 5’ AGAAGTGAATTCAATGGACGC 3’ |
|  | R, 5’ GCTGTCTAGATGGTGCTCAGCTCTC 3’ |
| derlin-1^∆^α | F, 5’ ACACGAGAATTCCACACAATGGAC 3’ |
|  | R, 5’ GTCTAGTCTAGAGAGTTTCGAGT 3’ |
| derlin-1^L204G^ | F, 5’ CGCCGCAGTTCGGGAAGCGCCTGGTGCC 3’ |
|  | R, 5’ GGCACCAGGCGCTTCCCGAACTGCGGCG 3’ |

*Underline indicates the changed codon.

**Primer sets used to generate modified pGEX-Derlin-1 constructs**

| Construct | Sequence |
| --- | --- |
| C-terminal of derlin-1 | F, 5’ AGTTCCAGAATTCGCAGGA 3’ |
|  | R, 5’ TCAGCTCTCGAGTTCAGTTGCG 3’ |
| C-terminal of derlin-1-FLAG | F, 5’ CCGAATTCTCACACAATGGACG 3’ |
|  | R, 5’ GGCGATCTCGAGTCACTTGTCGTCATCT  TGTAGTCTCCGTTGCGACCCAAGGTCATGC 3’ |
| C-terminal of derlin-1^∆SHP^ | F, 5’ CGACCGAAAGTCCCTAGGGCCGGGGCA  TGAC 3’ |
|  | R, 5’ GTCATGCCCCGGCCCTAGGGACTTTCG  GTCG 3’ |
| N-terminal of derlin-1 | F, 5’ CCGAATTCTCACACAATGGACG 3’ |
|  | R, 5’ CGTCTCGAGTAGCGGGTGAA 3’ |

**Primer sets used to generate wild type and modified pET-TER94 constructs**

| Construct | Sequence |
| --- | --- |
| TER94 full length (1-801) | F, 5’ CCGAATTCATGGCAGATTCC 3’ |
|  | R, 5’ GCTCGAGCTAACTGTAAAGATC 3’ |
| N-L1 fragment of TER94 (1-204) | F, 5’ CCGAATTCATGGCAGATTCC 3’ |
|  | R, 5’ GCTCGAGTCAACCACCGATAT 3’ |
| D1-L2 fragment of TER94 (205-477) | F, 5’ TCCGAATTCTGCCGCAAG 3’ |
|  | R, 5’ GCTCGAGTCATCCGATGTC 3’ |
| D2-C fragment of TER94 (478-801) | F, 5’ TCCGAATTCGGTCTGGAG 3’ |
|  | R, 5’ GCTCGAGCTAACTGTAAAGATC 3’ |

**Primer sets used in RT-PCR**

| Targeting cDNA | Sequence |
| --- | --- |
| derlin-1 | F, 5’ ATGGACGCTGGCGTGTGG 3’ |
|  | R, 5’ GTTGCGACCCAA GGTCATGC 3’ |
| bip | F, 5’ GCATATTACTGGCCGTCGTGG 3’ |
|  | R, 5’ CGAATGGATGCAGGCTGGGC 3’ |
| rp49 | F, 5’ ATGACCATCCGCCAGCATACAGG 3’ |
|  | R, 5’ TTACTCGTTCTCTTGAGAACGCACG 3’ |
| derlin-2 | F, 5’ GAGAATTCGCCATGAATGCC 3’ |
|  | R, 5’ TTCTCGAGTTGCTATTGCGG 3’ |

**Target sites of pUAST-caspase RNAi constructs**

| Construct | Targeting sequence |
| --- | --- |
| drice | 5’ ACACAGTACAAGCTGGATAACA 3’ |
| dcp-1 | 5’ GGAAAATCGGGGCAGCTTTATA 3’ |
| decay | 5’ GGTTTGTTCTTGAGCGTCTTGC 3’ |
| dronc | 5’ GCAACAGTGTGGAGGGAAAAGA 3’ |
| dream | 5’ CAATGGGTCTCCAAATGAAATC 3’ |
